# Unraveling female communication through scent marks in the Norway rat

**DOI:** 10.1073/pnas.2300794120

**Published:** 2023-06-12

**Authors:** Guadalupe Gómez-Baena, Kieran C. Pounder, Josiah O. Halstead, Sarah A. Roberts, Amanda J. Davidson, Mark Prescott, Robert J. Beynon, Jane L. Hurst

**Affiliations:** ^a^Centre for Proteome Research, Department of Biochemistry and Systems Biology, Institute of Systems, Molecular and Integrative Biology, University of Liverpool, Liverpool L69 7ZB, United Kingdom; ^b^Mammalian Behaviour and Evolution Group, Department of Evolution, Ecology and Behaviour, Institute of Infection, Veterinary and Ecological Sciences, University of Liverpool, Leahurst Campus, Liverpool CH64 7TE, United Kingdom

**Keywords:** scent communication, female rat, clitoral gland, proteomics, MUP

## Abstract

Chemical communication by females remains poorly understood, with most attention focused on female advertisement of sexual receptivity to males or mother–offspring communication. However, in social species, scents are likely to be important for mediating competition and cooperation between females determining individual reproductive success. Here, we explore chemical signaling by female laboratory rats (*Rattus norvegicus*) to test i) whether females target their deployment of scent information differentially according to their sexual receptivity and the genetic identity of both female and male conspecifics signaling in the local environment and ii) whether females are attracted to gain the same or different information from female scents compared to males. Consistent with targeting of scent information to colony members of similar genetic background, female rats increased scent marking in response to scents from females of the same strain. Females also suppressed scent marking in response to male scent from a genetically foreign strain while sexually receptive. Proteomic analysis of female scent deposits revealed a complex protein profile, contributed from several sources but dominated by clitoral gland secretion. In particular, female scent marks contained a series of clitoral-derived hydrolases and proteolytically truncated major urinary proteins (MUPs). Manipulated blends of clitoral secretion and urine from estrus females were strongly attractive to both sexes, while voided urine alone stimulated no interest. Our study reveals that information about female receptive status is shared between females as well as with males, while clitoral secretions containing a complex set of truncated MUPs and other proteins play a key role in female communication.

## Significance

Female scent communication achieves far more than that required to advertise estrus state to males. Female rats are intensely interested in gaining scent information about conspecific females and their receptivity. Further, the proteome of scent marks deposited by female rats and the targeting and uptake of scent information by other females reveal previously unknown layers of complexity in female rat semiochemistry. The intense interest in female scents by both the same and opposite sex will be of broad interest to the large community using this model species for basic biological and biomedical research. Their accessibility makes the rat an ideal model to further investigate the molecular and cognitive mechanisms that underpin complex female social signaling.

Chemical signals mediate sexual and social communication between conspecifics across a broad range of taxa (1). As scents can be deposited in the environment, they can provide information in the absence of the scent donor, with scent-marking behavior conspicuous in many terrestrial vertebrates (1, 2). Studies have focused particularly on scent marking by males, which often invest heavily in competitive scent signals that are used in both intramale competition and for advertising to potential mates (3–5). While often less conspicuous in terms of frequency, chemical investment, and behavior, females also emit signals through scent marks that are used to advertise estrus status to males, maternal communication with offspring, and for territorial marking (6). However, much less attention has been paid to the potential roles of scents in communication between females in social groups, even though there is increasing recognition that intrafemale competition is a very important determinant of female reproductive success. While females can cooperate with group members to gain access to resources and compete with rival groups, they also compete internally with group members for reproductive opportunities and for access to high-quality mates (7–10). Scents are likely to be important for signaling social dominance or quality between females (6, 8, 11) and have been linked to reproductive synchrony or suppression in some females that breed cooperatively (6).

Laboratory rodents have provided key models to unravel the information encoded in complex mammalian scent signals and to understand the neural pathways involved in processing scent information (12, 13). Laboratory mice and rats both derive from social species in which females both cooperate and compete with each other (8, 14–17), but very few studies to date have attempted to address the functions and mechanisms of intrafemale scent communication.

Wild Norway rats (*Rattus norvegicus*) typically live in large colonies in which multiple females share a burrow system and feeding sites while largely tolerating each other but are intolerant of females from neighbor colonies (15, 16). In addition to socially transmitted food preferences (18), cooperative foraging behavior between females is mediated through airborne scents (19). Females in estrus will solicit matings from multiple males in the colony and may cooperate with other estrous colony females to gain increased male copulatory stimulation while also competing for ejaculates from the most dominant males (20–22). As only some females in the same colony reproduce successfully (23), this suggests that intrafemale competition has a strong impact on female reproductive success, although female rats show little overt aggression (15, 24). We predict that female rats advertise information about their genetic identity and reproductive status to other females as well as to males and that females will be strongly motivated to gain information about other females from their scents.

Most studies of chemical signals in terrestrial mammals focus on airborne volatiles that can be detected through the main olfactory system (1, 25). However, studies of laboratory rodents have also highlighted the importance of proteins and involatile low-molecular-weight compounds, including steroid hormones in scents. These are detected through the vomeronasal system via nasal contact with the scent source (26). This secondary olfactory system, shared by most terrestrial vertebrates, has evolved receptors that are fine tuned to detect organic volatiles, proteins, and steroids in scents that have particular biological meaning, especially conspecific pheromones (27). In the context of proteins, rats and house mice (*Mus musculus*) invest heavily in a family of major urinary proteins (MUPs) that are produced in the liver and excreted at high concentration in urinary scent marks (28–30). MUPs bind small hydrophobic volatile molecules in a central pocket and can control the release of these volatile signals from scent marks (31–33). MUPs are also detected directly through specific vomeronasal receptors on nasal contact (34, 35). In house mice, the pattern of MUP isoforms in urine scents is under genetic control in both sexes, signaling individual and kinship identity (35–37), while the male MUP darcin acts as a male sex pheromone that attracts mates (38–40). Male rats also produce a high concentration of MUPs in their urine, but, unlike mice, these are almost absent from female rat urine (30, 41). Also in contrast to mice, observational studies indicate that rat scent marks consist of a mixture of urine and sebaceous secretions deposited when rats drag the genital region against the substrate or raised objects (24, 42). While the molecular components of naturally deposited female rat scent marks have not been well characterized, adult rats of both sexes have a pair of well-developed specialized sebaceous glands situated subcutaneously between the abdomen and the pubic skin (clitoral glands in females and preputial glands in males), with ducts that exude glandular secretions to the outside of the clitoris or prepuce (43). In contrast to female urine, MUPs are highly expressed in female clitoral glands (44, 45) and thus have the potential to be secreted in female scent marks.

Here, we use laboratory rats (*R. norvegicus*) to address key questions about female scent communication. First, do females target their deployment of scent information differentially according to their sexual receptivity and the genetic identity of both female and male conspecifics signaling in the local environment, consistent with information communicated to both sexes? Second, are females strongly attracted to gain the same or different information from female scents compared to males? To address this, our study integrates behavioral tests to assess the social context in which females deploy scent signals, and the attraction of males and females to female scent marks according to the donor’s receptivity, with detailed molecular characterization of proteins in female scent deposits. This reveals intense female interest in scents from sexually receptive females, while female scent proteins show a surprising level of complexity, contributed from clitoral secretions, urine, and other sources. Information provided by clitoral components is essential for the attraction of both males and other females, but females were attracted only when scents contained a full mixture of information from clitoral and urinary components, using different scent information to males.

## Results

### Scent Marking by Female Rats.

**Fig. 1.**
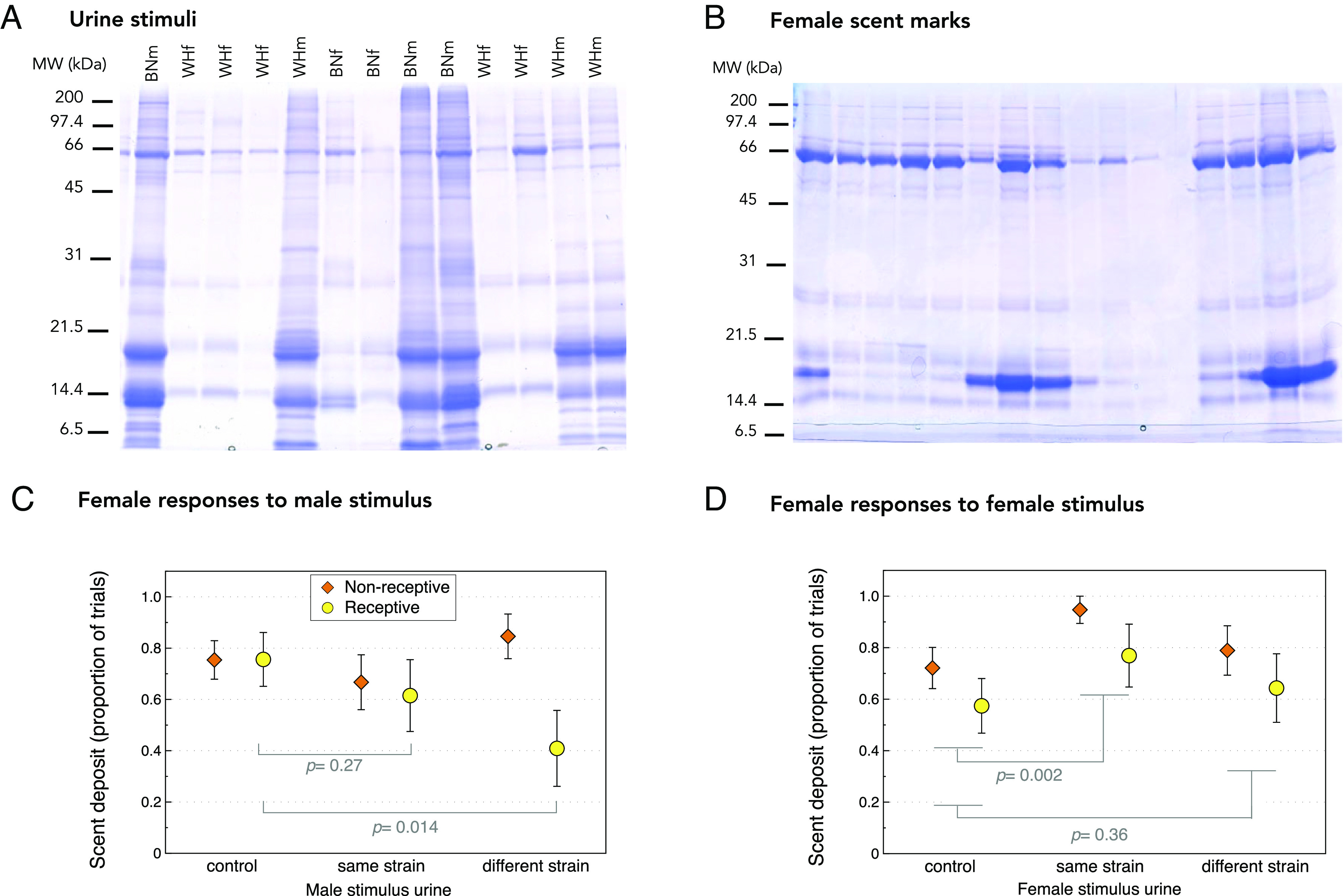
Scent marking by female rats. Example protein profiles of urine used as stimuli (BNf = Brown Norway female; BNm = Brown Norway male; WHf = Wistar Han female, and WHm = Wistar Han male) (*A*) and scent deposits from one Wistar female subject across 16 tests (*B*). Proportion of trials with visible scent proteins recovered from glass tiles or not (mean ± SEM for 10 Wistar Han and 10 Brown Norway females) in response to male urine or control water (*C*), and female urine or control water (*D*). Females were classed as sexually receptive (in estrus or proestrus, yellow circles) or nonreceptive (orange diamonds) by vaginal smear after testing. Data were analyzed by binomial generalized linear mixed-effects models (Table 1); *P* values show planned contrasts between each stimulus type and matched controls run on the same day.

As a first step to understand whether female rats deploy scent marks to communicate with both male and female conspecifics in their local environment, we assessed female scent marking in response to urine from unfamiliar males and females (Fig. 1). We used rats from two genetically distinct laboratory strains (Wistar Han and Brown Norway) to assess responses to unfamiliar donor scents from the same or different strain (representing animals genetically similar to the subject’s family or unrelated intruders). Females (10 per strain) were presented with male or female urine (Fig. 1*A*) from the same or different strain in a series of trials, conducted in randomized order in clean arenas. To assess whether a female’s estrous state (sexually receptive or not) influenced its scent-marking response, each female was tested twice with each stimulus type but different donors in trials conducted 2 d apart so that females would be in different stages of the estrous cycle. For each trial, a matched control test with no stimulus present was run on the same day. Scents deposited on two glass tiles on the floor of the arena were recovered by swabbing and proteins in the deposits visualized by Sodium Dodecyl Sulfate PolyAcrylamide Gel Electrophoresis (SDS-PAGE) (Fig. 1*B*). Binomial generalized linear mixed-effects models assessed whether the type of urine stimulus in the arena influenced the likelihood that females deposited scent marks and whether this depended on the female’s estrous state. Females were classified as “sexually receptive” (in estrus or proestrus) or “nonreceptive” (in metestrus or diestrus). All female urine donors were sexually receptive when stimulus urine was collected.

Females did not deposit scent on the glass tiles in every trial (Fig. 1 *C* and *D*). The likelihood of scent deposition differed between male and female stimuli and also according to the female’s sexual receptivity (interaction between receptivity, stimulus presence, and sex of donor: *P* = 0.020; interaction between stimulus presence and donor sex, *P* = 0.006; Table 1a). As females responded differently to male and female stimuli, we examined their sex-specific responses separately in more detail.

**Table 1.** Factors influencing the likelihood that female rats deposited scent marks in trials

Effect^*^	χ^2^	df	*P*
(a) All scent-marking trials			
Subject receptivity × urine presence × donor sex	11.72	4	0.020
Urine presence × donor sex	7.67	1	0.006
(b) Male stimulus trials			
Subject receptivity × urine presence × donor strain	9.95	4	0.041
Subject receptivity × urine presence	4.92	1	0.027
(c) Female stimulus trials			
Urine presence	7.24	1	0.007

^*^Binomial generalized linear mixed-effects models, with subject identity included as a random factor. Only significant interactions or main effects are shown.

When responding to male stimuli and matched control trials (Fig. 1*C*), scent marking depended on both sexual receptivity and whether the stimulus donor was from the same or different strain from the female (interaction between receptivity, stimulus presence, and donor strain: *P* = 0.041; interaction between female receptivity and stimulus presence, *P* = 0.027; Table 1b). Sexually receptive females were less likely to scent mark in the presence of male stimuli than in matched control trials (χ^2^ = 6.50, 1 df, *P* = 0.011; Fig. 1*B*). Receptive females suppressed scent marking significantly if the stimulus was from a different strain male (χ^2^ = 6.04, 1 df, *P* = 0.014), but a tendency to reduce scent marking if the stimulus was from a same strain male was not significant (χ^2^ = 1.23, 1 df, *P* = 0.27). By contrast, females in a nonreceptive stage of the estrous cycle were equally likely to deposit scent whether a male stimulus was present or not (Fig. 1*C*).

Female urine stimuli increased the likelihood that females deposited scent compared to matched control trials (*P* = 0.007; Table 1c and Fig. 1*D*). This was not influenced by the subject’s sexual receptivity (nonsignificant interaction between receptivity and urine presence: χ^2^ = 0.77, 1 df, *P* = 0.38). This increase in scent marking was mostly a response to urine from females of the same strain (χ^2^ = 9.16, 1 df, *P* = 0.002; Fig. 1*D*) as a slight increase when urine was from a different strain did not approach statistical significance (χ^2^ = 0.85, 1 df, *P* = 0.36). Thus, females were sensitive to the genetic background of female scent donors and were stimulated to signal when the female donor was from a very similar genetic background to themselves but not if they were from a foreign strain.

### Molecular Composition of Female Rat Scent Marks.

To understand the molecular basis of female communication, we analyzed the protein content of female scent deposits that were swabbed from glass tiles. In addition to scent marks from laboratory females, we also collected some scent marks deposited on glass tiles by two free-living wild female rats from a nearby farm (*Materials and Methods*).

**Fig. 2.**
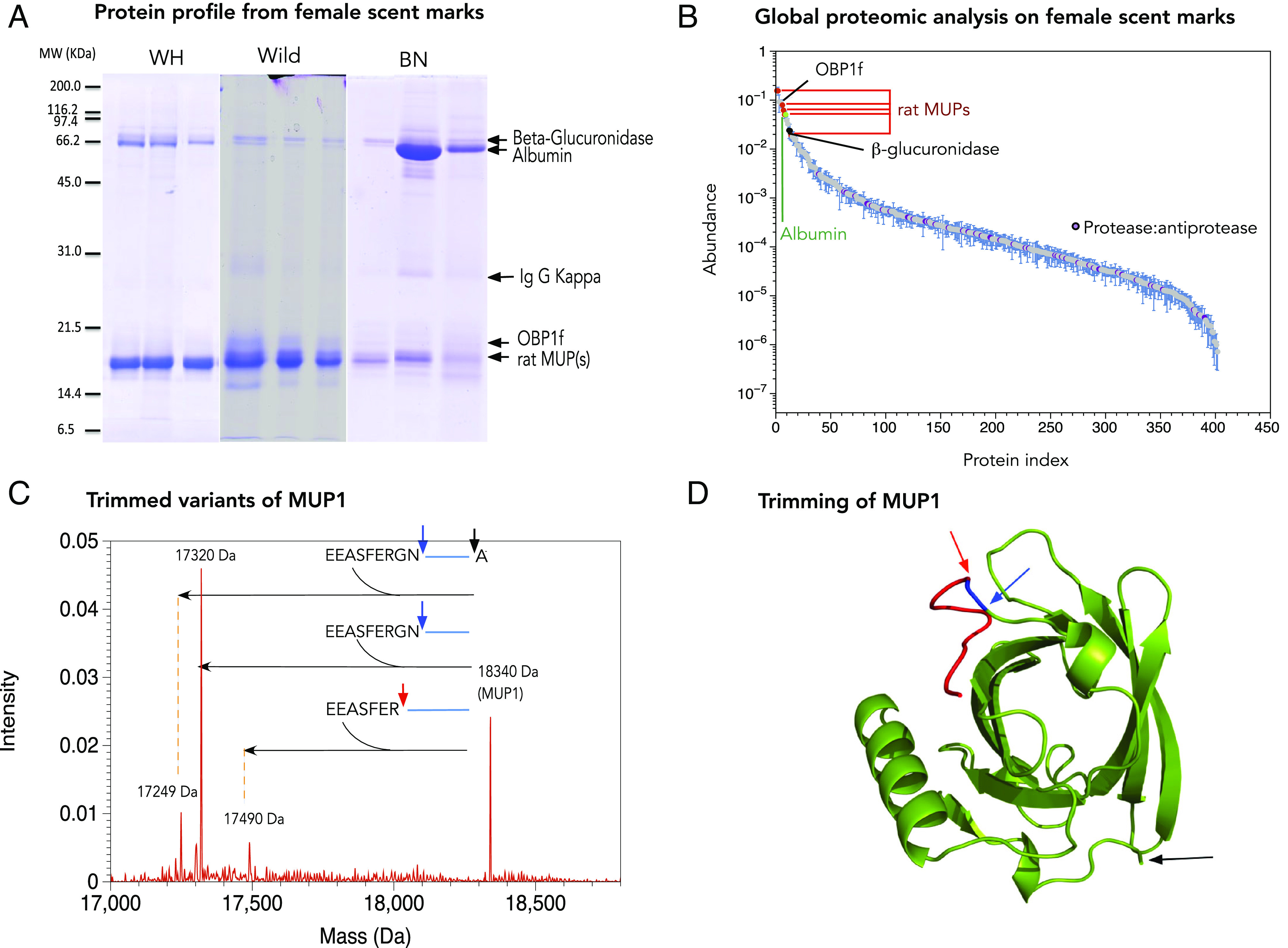
Protein profile of female rat scent marks. Female rat scent marks were recovered by swabbing and analyzed by SDS-PAGE from Wistar Han (WH), wild rats (Wild), and Brown Norway (BN). The major bands were excised and analyzed by PMF; band identities are indicated on the right-hand side of each gel (*A*). Female rat scent marks were analyzed by label-free quantitative proteomics. The quantitative profile, averaged across all samples (with error bars denoting SEM), covered six orders of magnitude. Key proteins: MUPs, beta glucuronidase, and protease/antiprotease protein are highlighted (*B*). The proteins in scent marks were also analyzed by electrospray ionization mass spectrometry to recover intact protein masses, revealing to a family of masses around 17 kDa, readily reconciled to partial trimming of MUP1 (*C*). The sites of trimming are readily accessible in a flexible unstructured region at the N terminus [*D*, based on a predicted model of MUP1; built using the Phyre (46)].

First, the scent protein fractions were visualized by SDS-PAGE (Fig. 2*A*). Differences were immediately apparent in the protein complement of scent marks compared to naturally voided rat urine [Fig. 1*A*, (30)]. The protein pattern of most scent marks included an intensely stained band migrating at 17 kDa (Fig. 2*A*), absent from voided female urine [Fig. 1*A*, (30)]. The strongly stained bands in scents were excised from gels, digested with endopeptidase LysC, analyzed by peptide mass fingerprinting (PMF), and searched against a database containing all predicted rat MUPs (as compiled in ref. 30). PMF profiles of the 17-kDa band matched to multiple isoforms of the rat MUP family (*SI Appendix*, Fig. S1). Digests of this band using trypsin to produce shorter peptides were analyzed by liquid chromatography–tandem mass spectrometry. This identified unique peptides matching all predicted MUP isoforms, except MUP 15, indicating that female scent marks contain a complex mixture of rat MUPs (Dataset S1). PMF of the 80-kDa band following LysC digestion revealed peptides unique to the enzyme β-glucuronidase (*SI Appendix*, Fig. S2*A*). Finally, PMF analysis of a relatively faint 20-kDa band revealed peptides unique to an odorant-binding protein OBP 1f (Uniprot KB Q9QYU9) (*SI Appendix*, Fig. S2*B*).

To provide a more complete definition of the total protein content of scent deposits, we conducted a global proteome analysis based on in-solution trypsin digestion of 24 scent deposit samples (seven from five Brown Norway females, nine from six Wistar Han females, and eight samples recovered from two wild rat females). A total of 469 proteins were identified at a false discovery rate of 1% and requiring at least two unique peptides identified per protein (Dataset S2). Label-free quantification confirmed the dominance of MUPs, β-glucuronidase (Uniprot KB F1LQQ8), and OBP 1f (Uniprot KB Q9QYU9) (Fig. 2*B*). Protein abundances spanned between five and six orders of magnitude. The protein distribution in scents obtained from Brown Norway, Wistar Han, and wild females showed an overall consistency, with pairwise comparisons of scent marks from all three sources maintaining a similar dynamic range (*SI Appendix*, Fig. S3).

In addition to the dominant proteins, we identified several other members of the lipocalin protein family in female scent samples that might play a role in scent communication, including several lipocalin-like proteins (Uniprot KB A0A096MJW3, A0A096MJR3, and F1M6Y6), OBP 2a (lipocalin 13, Uniprot KB B3EY84), OBP 2b (Uniprot KB A0A0G2JZ62), and the rat homolog of feline protein Fel D 4 (Uniprot KB B3EY86).

Additionally, scents contained several proteins that are specific to urine, including rat urinary proteins RUP-1, RUP-2, and RUP-3, which previously had been identified in rat urine (30). These are neither MUP nor OBP-like and have unknown function. Similarly, uromodulin, considered to be specific to urine, was also present in some scent marks.

### MUPs in Female Scent Marks Are Truncated.

Profiling of proteins in female scent deposits by intact mass Electrospray Ionization Mass Spectrometry (ESI-MS) revealed a cluster of multiple masses around 17 kDa (Fig. 2*C*) (30). The most prominent mass peaks in most samples were at 17,490 Da, 17,320 Da, and 17,249 Da (Fig. 2*C* and *SI Appendix*, Fig. S4). A few scent samples also displayed a larger mass corresponding to the nonphosphorylated form of MUP 1 [18,340 Da in Brown Norway and wild rats and 18,553 Da in Wistar Han rats (30)], suggesting that the smaller mass peaks could be truncated forms of MUP 1. Indeed, the intense masses observed at 17,490 Da and 17,320 Da can be predicted by excision of seven and nine N-terminal amino acids of MUP 1, respectively (Fig. 2*C*). The mass at 17,249 Da can be predicted by excision of nine N-terminal amino acids and one C-terminal amino acid of MUP 1 (Fig. 2*C*). These data are strong evidence for posttranslational truncation of MUPs to explain the high degree of polymorphism observed in mass peaks between 17 and 18 kDa (Fig. 2*C*). Further fragment masses are consistent with trimming of other MUPs present in high concentrations in scent marks, including MUP13 (*SI Appendix*, Fig. S4). Indeed, in virtually all scent marks, no masses were detected that correspond to intact rat MUPs. We confirmed the hypothesis that these proteins could be posttranslationally truncated forms by identifying peptides that correspond to the trimming of the N-terminal and C-terminal of MUP 1 in our proteomics analysis of female scents (Fig. 2*D*).

There was no correlation between intact mass MUP profile and rat strain (*SI Appendix*, Fig. S4), suggesting that posttranslational MUP truncation does not reflect genetic differences between females. Instead, each scent deposit had a complex pattern of mass peaks that we were unable to align to strain. Several endopeptidases and exopeptidases able to catalyze this trimming of MUPs were identified in female scent deposits (Dataset S2), including meprin and kallikrein, dipeptidyl peptidase 1 (Uniprot KB P80067), dipeptidyl peptidase 2 (Uniprot KB Q9EPB1), dipeptidyl peptidase 4 (Uniprot KB A0A0G2JTXS), aminopeptidase N-like (Uniprot KB A0A0G2JVE6), Xaa-Pro aminopeptidase (Uniprot KB Q99MA2), and S10-like carboxypeptidase (Uniprot KB Q6AYS3).

### Origin of Proteins in Female Scent Deposits.

The near-absence of MUPs in female urine contrasts with their dominance in scent marks, arguing strongly against a hepatic origin of these proteins, introduced to scent deposits via urine. MUPs are highly expressed in rat clitoral glands (44, 45), and this gland is a prime candidate for the MUPs in female rat scents. Further, β-glucuronidase is also highly expressed in rat clitoral glands (47, 48) and is also present in abundance in scent deposits.

To establish the clitoral gland as a major source of specific proteins observed in female scent deposits, we confirmed that MUPs in clitoral gland secretions are equivalently truncated. Intact mass analysis shows a very similar pattern to scent deposits (*SI Appendix*, Fig. S4).

**Fig. 3.**
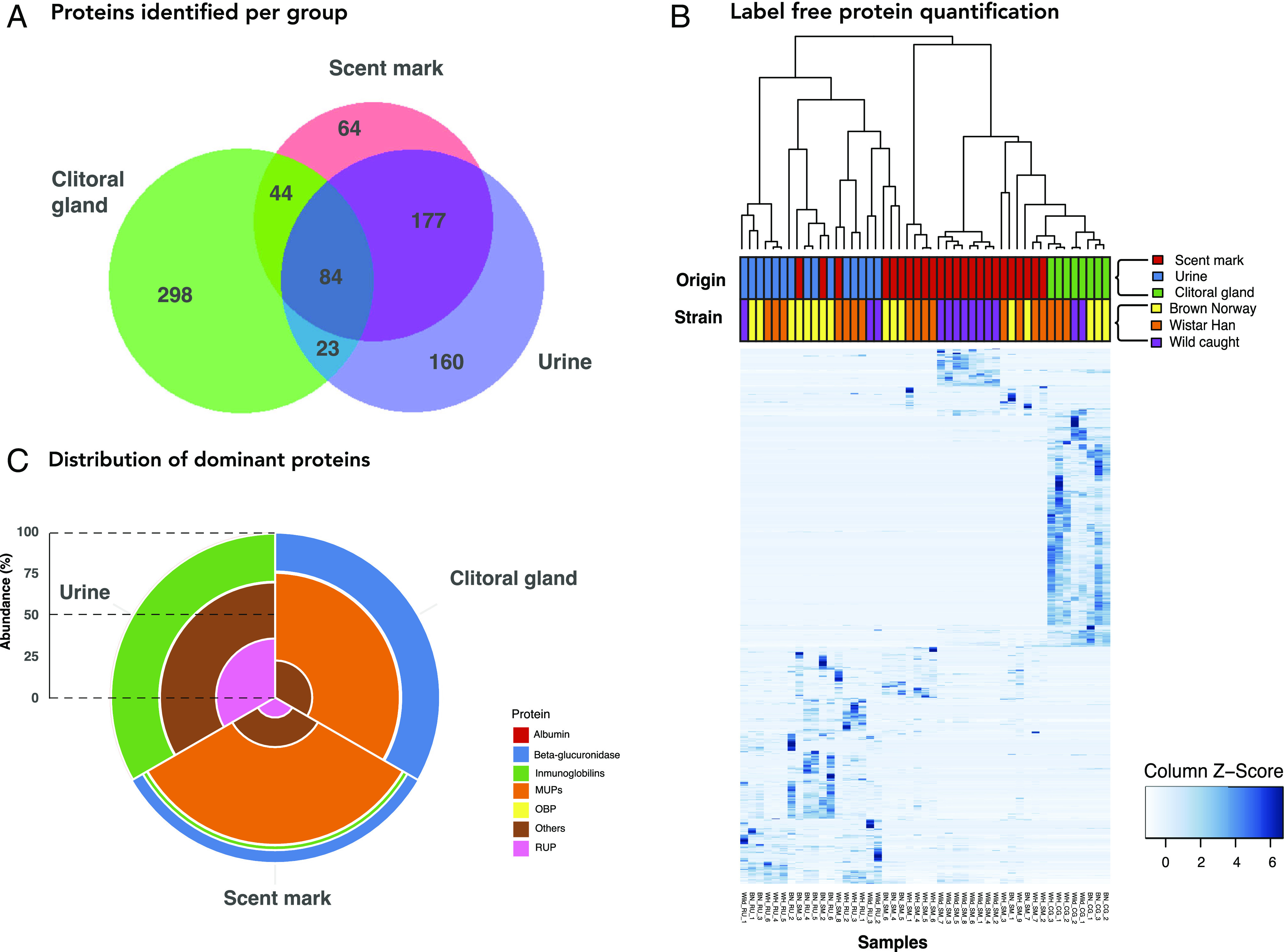
Proteomic analysis of tissue origins of scents. Female rat scent marks, urine and clitoral gland secretions from Brown Norway (n = 16), Wistar Han (n = 18), and wild rats (n = 13) were analyzed by label-free quantitative proteomics. Shared and unique proteins could be enumerated (*A*). The quantitative protein profiles were used to generate heat maps with hierarchical clustering using “average” as a clustering method and “Pearson correlation” as a measure of distance on normalized protein abundance (*B*, sample type and rat source indicated by colored bars at top). For the most abundant protein groups, a radial distribution plot emphasizes the distinction between the three sources (*C*).

To evaluate the proportional contributions of clitoral gland secretion and urine to the protein composition of scent deposits, a second label-free quantitative proteomic analysis compared scent marks, clitoral extracts and urine from Wistar Han, Brown Norway, and wild-caught female rats (Fig. 3). A total of 930 proteins were identified at 1% false discovery rate, with a minimum of two unique peptides for identification, with a total of 506 protein groups in clitoral gland secretion, 461 protein groups in urine, and 469 protein groups in scent mark samples (Dataset S3). Most proteins in scent deposits were identified in urine and/or clitoral extracts, with 177 out of 469 scent proteins (38%) exclusive to urine and 44 (9%) exclusive to clitoral extract (Fig. 3*A*). We used the label-free quantitative data to compare the normalized profile of proteins in scent marks, urine and clitoral gland extracts (Fig. 3 *B* and *C*). The average proportional abundance for each protein in samples from the same origin (clitoral extract, voided urine, scent marks) identified the most abundant proteins per sample type (Fig. 3*C* and *SI Appendix*, Fig. S5). Several MUP isoforms and the enzyme β-glucuronidase were among the most abundant proteins in both clitoral extracts and scent marks across all three rat strains (Brown Norway, Wistar Han, and wild females), supporting the hypothesis that clitoral glands are the major source of female scent mark protein. Scent marks also contained a large number of proteins derived from urine, including RUPs 1 to 3 (the most abundant proteins in female urine, not to be confused with MUPs), albumin, uromodulin, and cystatin C (*SI Appendix*, Fig. S5). Interestingly, cystatin C is absent from scent marks and from the urine of wild rats, which might imply better renal health compared to laboratory strains (49).

We identified a further 64 proteins in female rat scent marks that were absent in urine or clitoral glands extracts and which thus may originate from other sources. Some of these have been described in the ventral lobe of male rat prostate, including cystatin-related protein 1 (UniProt KB A0A0G2JSU6), prostatic steroid-binding proteins C1 (UniProt KB P02782), and C2 (UniProt KB P02781) (50, 51). These proteins were identified only in samples from wild-caught female rats.

### Variable Inclusion of Clitoral Gland Secretion in Scent Marks.

To establish whether females added clitoral gland secretions to scent marks differentially when responding to male or female urine stimuli compared to matched controls, or varied this according to sexual receptivity, we examined whether deposits recovered in female scent-marking trials contained SDS-PAGE bands corresponding to MUPs. An MUP band was apparent in 58 ± 3% of scent deposits recovered across all scent-marking trials. Females did not appear to actively modify this according to scent-marking context, with no significant effects of female receptivity, or the presence of male or female urine from the same or different strain donors on the proportion of scent marks containing clitoral proteins. The only factor that influenced this was the genetic strain of the scent-marking female. Clitoral secretion was present in 69 ± 5% of scent deposits from Brown Norway females, but in only 48 ± 7% of scent deposits from Wistar Han females (χ^2^ = 4.78, 1 df, *P* = 0.029). The small number of samples from wild female rats all contained clitoral secretions. The lower proportion of scent marks from Wistar Han females that contained clitoral secretions could have been influenced by artificial selection on this strain in the laboratory.

### Attraction to Female Rat Scent Marks.

To assess the attraction of other rats to female scent marks containing clitoral secretions, we generated a randomly assorting hybrid cross between Wistar Han and Brown Norway laboratory strains. This ensured that individual subject and scent donors were genetically unique with individual-specific scents, more closely modeling variation between individuals in natural populations. We examined the attraction of male and female rats to scent marks from an unfamiliar female conspecific and assessed whether attraction was influenced by the donor’s sexual receptivity. Scent mark stimuli were naturally deposited on a clean petri dish lid while individual female donors explored a clean arena. We then compared the bias in time that individual subject rats spent investigating female scent marks relative to a clean petri dish lid streaked with water while exploring another clean arena. To test whether attraction was influenced by nasal contact with female scent compared to airborne odor alone, stimuli were presented behind a mesh barrier that either allowed nasal contact (surface held against the mesh) or not.

**Fig. 4.**
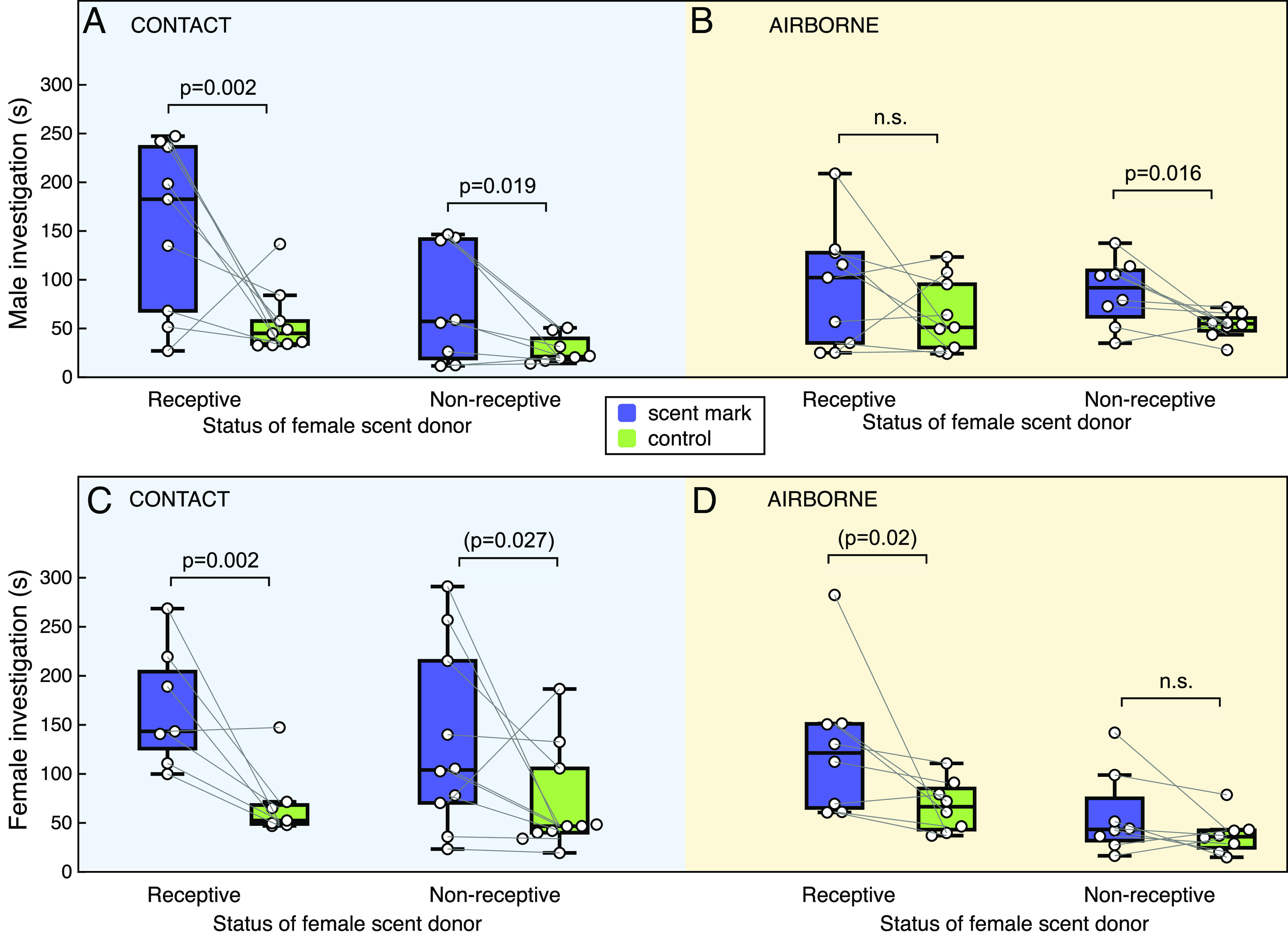
Attraction to female rat scent marks. Duration of investigation of a scent marked (purple box) vs. clean (green box) petri dish lid while exploring a clean arena by male (*A* and *B*) and female (*C* and *D*) hybrid rats (boxes show median and interquartile range; circles show individual data points). Scents, presented behind a mesh barrier, could be contacted (*A* and *C*) or not (*B* and *D*), with 8 to 10 rats per test according to availability. Lids were naturally scent marked by sexually receptive or nonreceptive females exploring a clean arena. Repeated-measures ANOVAs assessed the effects of donor receptivity and scent contact on investigation of female scent vs. control by each sex (Table 2). *P* values in the figure show post hoc matched-pair *t* tests assessing greater attraction to scent than control for each separate stimulus (values in parentheses not significant when Holm–Bonferroni correction applied for multiple comparisons).

Female scent marks were attractive to males, with most males investigating female scent more than the control stimulus (bias in sniffing and gnawing at the mesh covering the scent mark vs. control stimulus, *P* < 0.0001; Table 2a and Fig. 4 *A* and *B*). The strength of male attraction was influenced by their ability to contact the scent and by the sexual receptivity of the female donor (significant interactions between scent bias, contact, and donor receptivity, Table 2a). Attraction was much stronger when males could contact female scent marks (interaction between scent bias and contact, *P* = 0.019) and when the scent contacted was from a sexually receptive donor (interaction between donor receptivity and contact: *P* = 0.04, Fig. 4*A*). Airborne scent alone was more weakly attractive to males, regardless of a donor’s receptivity (Fig. 4*B*). Thus, information gained on contact appears important to stimulate strong male attraction specifically to scents from sexually receptive females, although all female scents stimulated some male investigation.

**Table 2.** Sex-specific attraction to female scent marks

Effect^*^	Partial η2	F	df	*P*
(a) Male attraction to female scent marks^†^				
Attraction to scent vs. control	0.499	28.88	1,29	<0.0001
Donor receptivity	0.208	7.60	1,29	0.010
Contact with stimulus	0.008	0.23	1,29	0.63
Attraction × contact	0.176	6.18	1,29	0.019
Donor receptivity × contact	0.134	4.51	1,29	0.042
Attraction × receptivity × contact	0.112	3.67	1,29	0.065
(b) Female attraction to female scent marks				
Attraction to scent vs. control	0.446	24.13	1,30	<0.0001
Donor receptivity	0.237	9.32	1,30	0.005
Contact with scent	0.181	6.61	1,30	0.015
No significant interaction terms				
(c) Female vs. male attraction to female scent marks				
Contact × subject sex	0.095	6.22	1,59	0.015
Subject sex × contact × donor receptivity	0.110	7.33	1,59	0.009

^*^Effects of subject sex, scent donor sexual receptivity, and scent contact on time spent investigating female scent marks vs. a control stimulus (repeated-measures ANOVA). The response variables for female subjects (b) and for the comparison between male and female subjects (c) were log transformed to meet the assumptions of parametric analyses (residuals approximated a normal distribution). Nonsignificant interactions were removed from models.

^†^One male given contact with scent marks from a receptive female was a strong outlier, spending much more time with the water control than with female scent marks (Fig. 4*A*). This outlier was removed from the models in (a) and (c) to meet assumptions for parametric analysis. As videos were assessed blind to scent location, we are unable to resolve whether this male’s response was a true outlier or an experimental error.

Female scent marks were also attractive to other females (*P* < 0.0001, Table 2b and Fig. 4 *C* and *D*). Indeed, the level of attraction shown by female and male conspecifics was very similar (Fig. 4), with no overall sex difference in the bias for investigating female scent over a control stimulus (no interaction between subject sex and scent bias, *P* = 0.90). Like males, females were more strongly attracted to scent marks from an unfamiliar female if the donor was sexually receptive (*P* = 0.005) and they could contact the scent (*P* = 0.015; Table 2b). However, scent contact and donor receptivity affected females and males slightly differently (interaction between subject sex, scent contact, and donor receptivity, *P* = 0.009; Table 2c). Females were able to discriminate another female’s receptive state based on airborne odors alone and showed very little interest in airborne odors from nonreceptive females if they could not contact the scent source (Fig. 4*D*). By contrast, males showed a low level of attraction to airborne odors from female scent marks whether the female was in a receptive or nonreceptive state (Fig. 4*B*). For both sexes though, contact with female scent marks provided additional information that strengthened attraction to scent marks from receptive females, with rats spending a prolonged 160 ± 19 s investigating receptive female scents during a 600-s test.

### The Impact of Clitoral Secretion on Attraction to Female Scent Marks.

As female scent marks consist of a mixture of components from urine and secretions from other glands, in particular the clitoral gland, we tested male and female attraction separately to voided urine (no detectable clitoral contribution on SDS-PAGE), to clitoral secretion obtained from dissected clitoral glands, or to a mixture of these to establish whether clitoral secretions and/or urine components are responsible for male and female attraction to female scent marks.

**Fig. 5.**
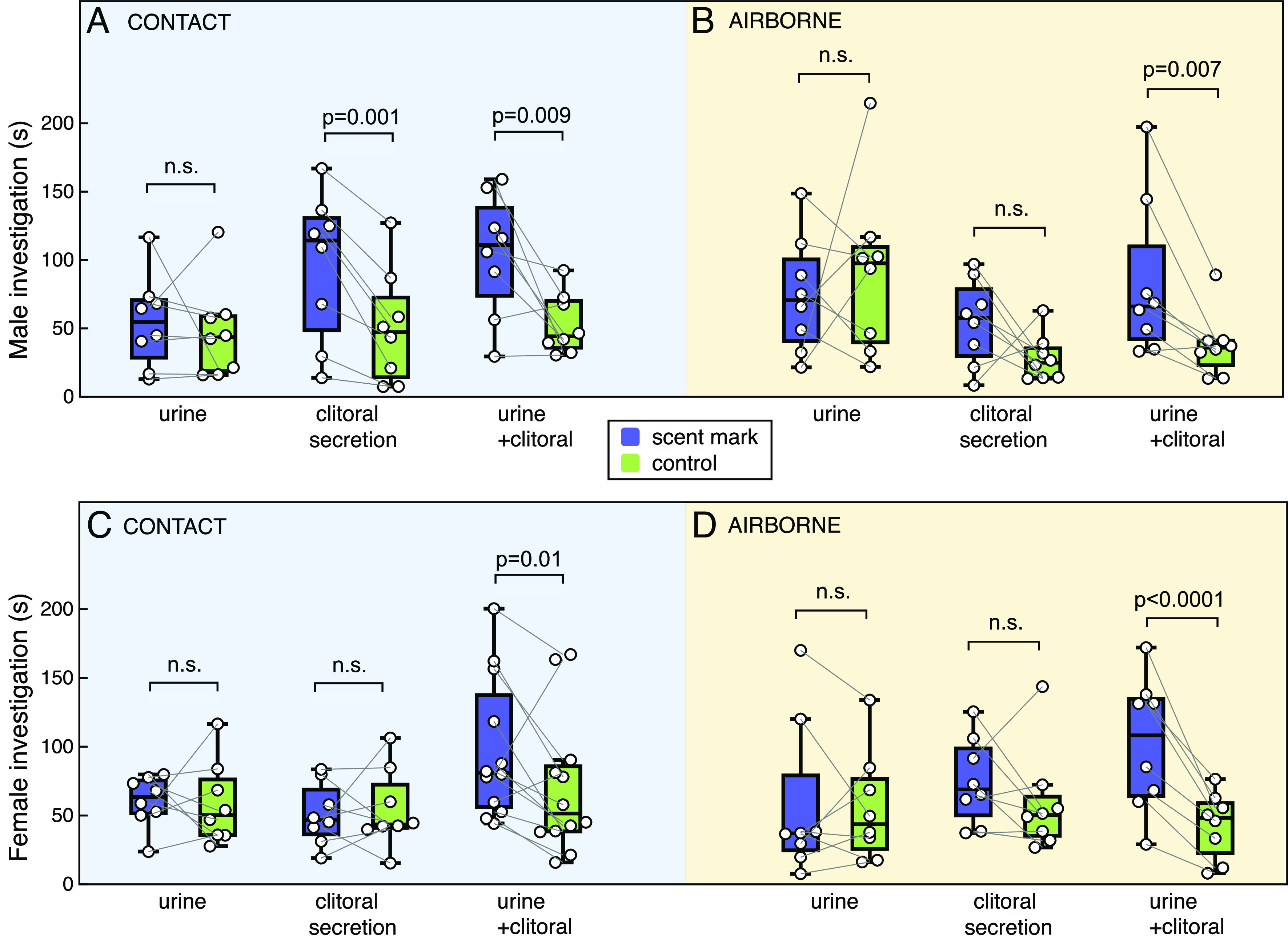
The impact of clitoral secretion on attraction to female scent marks. Duration of investigation of a scent stimulus (purple box) vs. water (green box) while exploring a clean arena) by male (*A* and *B*) and female (*C* and *D*) hybrid rats (boxes show median and interquartile range; circles show individual data points). Stimuli, presented on filter paper behind a mesh barrier, could be contacted (*A* and *C*) or not (*B* and *D*), with a minimum of eight rats per test. Scent stimuli were 50 μL of clitoral secretion, voided urine containing no visible clitoral proteins, or an equal mix. Repeated-measures ANOVAs assessed the effects of scent type and scent contact investigation of female scent vs. control by each sex (Table 3). *P* values on figure show post hoc matched-pair *t* tests assessing greater attraction to scent than control for each stimulus (all *P* values remain significant when Holm–Bonferroni correction applied for multiple comparisons).

Males were attracted to female scents that contained clitoral secretion but not to urine alone (Fig. 5 *A* and *B*; interaction between attraction and scent type: *P* = 0.01, Table 3a). Consistent with their response to naturally deposited scent marks, males were more strongly attracted to clitoral secretion or to clitoral secretion mixed with urine if they had nasal contact with the scent source, which increased their investigation of both the female scent and nearby control locations (interaction between contact and scent type, *P* = 0.021). However, males showed no attraction to voided female urine, regardless of their ability to contact the urine scent (Fig. 5 *A* and *B*).

**Table 3.** Sex-specific attraction to female scent mark components: Urine, clitoral secretion, or mixture

Effect†	Partial η2	F	df	P
(a) Male attraction to female scent components				
Attraction to scent vs. control	0.246	13.71	1,42	0.0006
Attraction × scent type	0.194	5.06	1,42	0.011
Contact × scent type	0.168	4.24	1,42	0.021
(b) Female attraction to female scent components				
Attraction to scent vs. control	0.195	11.16	1,46	0.002
Attraction × scent type	0.257	7.96	2,46	0.001
(c) Female vs. male attraction to female scent components				
Subject sex × scent type × contact	0.071	3.35	2,88	0.040

Females were attracted to a mixture of urine and clitoral secretion but not to urine or to clitoral secretion alone (Fig. 5 *C* and *D*; interaction between attraction and scent type, *P* = 0.001; Table 3b). Contrasts confirmed that females were more strongly attracted to a urine and clitoral secretion mix than toward urine alone (Bonferroni *P* = 0.003) or to clitoral secretion alone (*P* = 0.030). Female attraction was not influenced by their ability to contact the scent source in these tests, with strong attraction to a urine and clitoral secretion mixture whether they could contact the stimulus or not.

Overall, these tests show that both sexes were significantly attracted to odors emanating from a mixture of female urine and clitoral secretion, while males (but not females) were also attracted by contact with clitoral scent alone. Neither sex showed any significant attraction to voided female urine that contained no detectable clitoral secretion.

## Discussion

A focus on female communication in social species is long overdue. Female rats spontaneously deposited scents when exploring a familiar clean arena, but the presence of scents from other females was an additional stimulus to deposit scent. This was particularly the case when females encountered other females of their own strain, regardless of whether they were in a receptive stage of the estrous cycle or not. The stronger increase in scent marking in response to own strain compared to different strain female scent is consistent with motivation to communicate with other females from their own colony rather than a territorial response to unfamiliar female intruders. Nonetheless, as scents deposited in the local environment broadcast information to any animals in the vicinity, female scent marks may still play some role in communication between colonies.

Early studies suggested that scent marking by female rats strongly depends on their stage of the estrous cycle, with only infrequent marking by females until they approach estrus (52, 53), leading to assumptions that the main function of female scent marking must be to advertise estrous state to males. However, scent marking in these studies was assessed when females were placed in novel environments rather than marking their familiar home area (54). Here, we ensured that females were familiar with test environments, and each female was tested with multiple stimuli in different stages of their hormonal cycle all in the same environment. In agreement with other studies conducted in familiar test environments (54–56), female rats showed a similar high probability of scent marking whether receptive or not. As females continually deposit scents around their familiar area that contain information about their identity and current state of receptivity, this information is advertised to both males and females visiting the area.

We found no evidence that scents from males stimulated any increase in scent marking from estrous females. By contrast, females suppressed scent marking when they were sexually receptive in the presence of scent from males of a different strain. The mating behavior of Norway rats is such that female rats in estrus are pursed by multiple males in their colony when they leave their burrows. Females solicit copulations by approaching a male and, typically, will approach and copulate with multiple males from their own colony, while the males take turns to mount and inseminate the female (15, 22, 57, 58). Receptive females may show a degree of mate choice for own colony males by suppressing signals that would attract pursuit when they detected male scents from a genetically foreign strain, which they did not do in the nonreceptive phase of their estrous cycle. Estrous females also stay close to their burrow in the colony and have been reported to flee from neighboring males (15, 58). By contrast, active advertisement of a nonreceptive state through scent marks may help to minimize potential harassment from any males when females are not ready to mate.

Unsurprisingly, males were strongly attracted to investigate female scent deposits, particularly when they had nasal contact with estrous female scent, with median investigation duration almost doubling with scent contact. In addition to detecting airborne volatiles emanating from scents through the main olfactory system, nasal contact allows detection and processing of specific scent components through the vomeronasal system. Specific vomeronasal receptors allow detection of nonvolatile proteins and peptides including MUPs (35, 59, 60), female sex hormones excreted in urine that attract males (61, 62), and volatile sex pheromones that are delivered in solution to the vomeronasal organ. Such signals can then stimulate specific neural pathways through the vomeronasal system that are important for coordinating hormonal and behavioral responses to sexual stimuli (40, 63).

Importantly though, our tests reveal that females were just as intensely attracted to investigate scent marks from other females, particularly from a donor in estrus or proestrus, while scents from other own strain females stimulated them to increase their own scent marking. This confirms that females have a strong motivation to gain information from another female’s scent, especially when the donor is sexually receptive. Females discriminated between airborne odors from receptive and nonreceptive female scent marks, showing very little interest in nonreceptive female scent if they could not contact the source but greater persistence of investigation toward receptive female scent. Like males, their much more prolonged investigation of contacted scents suggests strong motivation to gain further information through the vomeronasal system. Further, prolonged investigation of sexually receptive female scents suggests that they were particularly driven to gain information about other potentially reproductively active females in the local area. Female advertisement of their individual identity and status through scent marks may play an important role in coordinating group mating within the colony, potentially mediating both cooperation between colony females and intrafemale competition for the highest quality mates. When groups of laboratory rats are allowed to mate in a seminatural habitat, females can regulate the timing of vaginal intromissions and ejaculation by approaching males to solicit copulations. Compared to mating rates controlled by males, the rate of vaginal stimulation paced by females is slower and more effective in inducing the neuroendocrine changes required for successful embryo implantation, sperm transport is enhanced, and the female’s estrous period abbreviated (20–22). If more than one female in the group is in estrus, the females cooperate and take turns for male intromissions. This increases male reproductive investment by stimulating more male copulatory behavior while also slowing the pace of mating for females, allowing females to optimize the interval between intromissions to stimulate a progestational state for successful implantation (21, 22). In addition to stimulating males to seek and mate with females, sharing and monitoring information on individual receptivity within the group via scent deposits could help females adjust their behavior according to the number and identity of females advertising current or impending receptivity. Although same-group females in estrus will cooperate and take turns to gain stimulatory intromissions from males, females also appear to compete with each other to gain ejaculates from the highest quality (dominant) males. McClintock et al. (22) showed that females were more likely to intercept a male that was following another female when the male was about to ejaculate, with some females consistently more successful at soliciting and intercepting males to receive ejaculates than others. Intense close-contact investigation of female scents may provide males with information about the physiological and/or genetic quality of estrous females that helps to influence their preference between soliciting females in this competitive scenario. Females might also use such information to compete with each other or adjust their mating strategy according to local competition to enhance their likely reproductive success. As yet, we have little understanding of the social dynamics involved in the complex group mating system that has evolved in this species and the roles that scent information plays in mediating individual mating strategies.

Characterization of the proteomes of natural scent deposits revealed a protein pattern in scent marks that differs greatly from voided urine. Female scent marks are dominated by lipocalins, notably MUPs, whereas these proteins are almost imperceptible in female urine. In addition, β-glucuronidase, an enzyme that cleaves glucuronide conjugates on many molecules, is very abundant in female scents. The proteomics analysis is consistent with both the MUPs and β-glucuronidase in scent marks deriving from clitoral glands, with female scent marks consisting of a mixture of clitoral secretion and urine in variable quantities. In rats, female clitoral glands are generally as well developed as preputial glands in males, and gland size does not appear to vary with estrus cycle (43). Males prefer the odor of intact over clitoralectomized female rats (64) and are attracted to airborne odor from a clitoral gland homogenate compared to a muscle and liver homogenate (65). Our tests with urine, clitoral secretion, or a blend confirm that the components of female scent marks that stimulate prolonged investigation by males are of clitoral origin. However, females were only attracted by a blend of clitoral secretions and urine, similar to the composition of natural female scent marks. Urinary components in scent marks appear to provided important information to other females, even though neither sex was attracted by voided female urine alone, which contains multiple volatile odorants that are enriched in adult females (66).

In contrast to urinary MUPs in house mice, clitoral MUPs in female rat scent marks are truncated. While the presence of MUPs in rat clitoral glands has been demonstrated previously in *R. norvegicus* (44, 45, 67, 68), and the black rat (*Rattus rattus*) (69, 70), the evidence for substantial truncation has not previously been revealed. What is the biological significance of this posttranslational complexity? The protein pattern was complex and variable, and did not align with strain, inconsistent with the idea of a combinatorial identity code that is evident in house mice (35, 37, 71). An alternative hypothesis is that truncation plays a role in ligand binding and release. Truncation is at the unstructured N terminus that caps the ligand binding pocket. Proteolysis could thus alter accessibility of the central calyx, altering binding or release of captive low-molecular-weight ligands. Further analysis is required to understand the effect of trimming on volatile binding and release, particularly as the natural ligands are as yet unknown. However, volatile odorants have been identified in female rat clitoral glands that stimulate extended investigation from both sexes and could be candidates (72). A second notable feature of clitoral gland secretion is the high-level release of β-glucuronidase. Gonadal steroids are excreted as glucosiduronate conjugates that are odorless and which have enhanced water solubility to aid secretion. β-glucuronidase is known to release steroids from steroid glucosides (73). Synthesis of rat β-glucuronidase seems to be under hormonal control, particularly estrogen and other hormones related to pregnancy and lactation (74). As a working hypothesis, it is possible to combine the action of β-glucuronidase, MUPs, and their proteolysis in a model of prepheromone secretion, hydrolysis, and MUP binding/release, adding dimensions of complexity that require further study (*SI Appendix*, Fig. S6).

Some proteins previously identified in male prostate glands were also identified in wild females, although the sample size was very small. Prostate-like glands (or Skene’s paraurethral gland) have been described in females from several mammal species, including Wistar Han (75) and Brown Norway rats (76). Skene’s paraurethral gland is morphologically similar to the ventral lobe of the male prostate (77), although morphologically, prostate gland presentation in female rats is heterogeneous, often unnoticed macroscopically, and not fully developed. Secretion from such glands has been detected (76, 77), and testosterone treatment in females promotes an increase in both gland size and secretion (77). The biological role of female prostate-like glands remains unclear, but the findings here suggest a putative role in scent communication.

In conclusion, our study shows that female rats communicate with other females through scent marks deposited around their familiar area regardless of their current estrous state. Prolonged investigation of scents from sexually receptive females suggests that females are particularly driven to gain information about other females in the local area that are sexually receptive. Female scent marks contain a complex mix of proteins, dominated by a variable set of truncated MUPs derived from the clitoral glands, but information communicated by the truncated MUPs remains to be determined. Our findings identify rats as a laboratory model for further studies to elucidate the molecular and cognitive mechanisms that underpin complex female social signaling.

## Materials and Methods

### Animals and Sample Collection.

Initial behavioral and molecular studies of scent marking and scent components used adult Wistar Han outbred rats and Brown Norway BN/RijHsd inbred rat strains originating from Harlan UK (now Envigo) and bred in-house. Subsequent studies examining responses to scent marks and scent components used adult rats from a hybrid in-house colony derived by initially crossing Wistar Han and Brown Norway strains and outbreeding offspring over subsequent generations (F3 to F7). All animals were housed in 56 × 38 × 25 cm cages (GPR2, North Kent Plastics) in single-sex small family groups or singles (one to four sisters or brothers per cage during the test period), on a reversed 12:12 h light cycle with lights off at 08:00 h. Tests were conducted during the active dark period under dim red lighting. Rats were maintained on Corn Cob Absorb 10/14 substrate with paper wool nest material and ad libitum access to water and food (Lab Diet 5LF2 EURodent Diet 14%, St Louis, MO, USA). Plastic tubes (8 cm diameter, Techniplast UK Ltd.), nest material, and cardboard shelters were provided for home cage enrichment. All animal care protocols were in accordance with the University of Liverpool Animal Welfare Committee requirements and UK Home Office guidelines for animal care. Urine was collected by placing a donor rat into a clean cage (56 × 38 × 25 cm, GPR2, North Kent Plastics) with a mesh bottom and collection tray placed below. Animals were left for 30 min, after which urine was collected and stored at −20 °C until use. All urine was collected 1 to 2 wk prior to testing and analyzed by SDS-PAGE if required. Rats were humanely killed and the clitoral glands dissected. Contents were expressed and the extracts resuspended in 100 μL phosphate saline buffer (PBS, 10 mM sodium phosphate buffer, pH 7.4, containing 0.154 M sodium chloride) before being stored at −20 °C until use.

To check that proteins in scents marks from laboratory females were similar to those of wild female rats, we collected a small number of scent marks from free-living females on a local farm (4 and 4 scent marks from two female donors). Glass tiles were placed out where wild rats were active overnight and all visits to tiles monitored using a Bushnell trail camera. A large plastic cylinder naturally scent marked by hybrid laboratory females was placed near one set of tiles and a clean cylinder near the other set of tiles. The sex of rats visiting and scent marking the tiles was confirmed both from recorded videos and from molecular differences in the urine and scent marks between male and female rats.

Urine samples from three adult wild females were provided by the former Central Science Laboratory of Defra (now part of the Animal and Plant Health Agency, UK) from rats trapped on farms within 15 miles of the Central Science Laboratory (Sand Hutton, North Yorkshire). Wild-caught animals were individually housed in suspended wire cages with free access to food and water. Urine samples were collected overnight on a clean waxed paper sheet in the tray under the cage. All samples were aspirated by pipette, avoiding feces and food fragments, and stored at −20 °C until use.

Clitoral gland extracts were obtained from two adult wild females caught from a farm in the North West and the other from Hampshire, UK. Female rats were humanely euthanized and the clitoral glands were dissected, and secretions were extracted by gently applying pressure to the gland. The secretions were resuspended in 50 μL of 50 mM ammonium bicarbonate before being stored at −20 °C until use.

### Scent-Marking Tests.

Scent-marking trials were run in clean white melamine arenas (60 × 70 cm). To stimulate scent marking, a urine stimulus (50 μL) was streaked onto a glass tile (100 × 100 × 5 mm) that was then secured vertically to the center of one wall of the test arena. An equivalent control tile streaked with water was secured similarly to the opposite wall of the arena. Two clean glass tiles were secured to the arena floor in front of each stimulus tile to collect any female scent deposited in a 10-min trial. Control trials were the same as urine stimulus trials except 50 μL deionized water was streaked on both stimulus tiles secured on opposite walls of the test arena. In both types of trials, scent marks deposited on the two clean floor tiles were recovered at the end of the trial by swabbing the surfaces with a cotton bud tip soaked with 50 μL of deionized water. To recover scents, swabs were centrifuged in an Eppendorf containing a hole in the base for 2 min at 13,000*g* such that liquid from the swab passed into an intact Eppendorf below.

In total, each female subject (n = 10 Wistar Han and n = 10 Brown Norway adult females) was assessed in a total of eight urine stimulus tests and eight matched control trials, run in a randomized but balanced order. Control trials were run on the same day as each urine stimulus trial, approximately 4 h before or after the urine stimulus test. Each female was tested with four different types of urine stimuli (unfamiliar male or female urine from own strain or different strain). To assess whether a female’s estrous state (sexually receptive or not) influenced its scent-marking response, females were tested with each type of stimulus in two trials conducted two days apart (using different unfamiliar scent donors) so that females would be in different stages of the estrous cycle. Estrous stage was assessed by vaginal swab conducted after trials for that day had been completed. To encourage normal female estrous cycling, females were primed with soiled male nest material 3 d prior to the start of tests. All female urine donors were in proestrus or estrus when stimulus urine was collected for these trials.

Recovered scent deposits from each trial were frozen until all trials had been conducted; then, proteins in deposits recovered from each tile and trial were visualized on SDS-PAGE (Fig. 1*A*). We recorded scent deposits as present if scent proteins were evident on either or both of the two swabbed tiles per trial or absent if no scent proteins were present on either tile.

### Bioassay of Attraction to Female Stimuli.

All testing was carried out during the dark phase of the 12 h:12 h light cycle under dim red lighting. Tests were conducted in a clean floor arena (120 × 120 × 81 cm, melamine) to which females or males were habituated for 1 h on the day prior to testing. To present stimuli during tests, clean plastic tubes (8 cm diameter, Techniplast UK Ltd.) were inserted through holes in the center of two side walls at right angles (holes were covered by opaque plastic plates during habituation). Mesh caps ensured that rats could not enter the tubes. To assess responses to scent marks deposited naturally by donors on the lid of a clean petri dish (55 mm diameter), a freshly scent-marked petri dish was presented inside one stimulus tube, held either against the mesh to allow full nasal contact or equivalently placed halfway along the tunnel to assess response to airborne volatiles only. A control petri dish streaked with 50 μL water was placed equivalently in the other stimulus tube. The position of the stimulus was randomized but balanced to ensure that an equal number of each stimulus type was presented on each side. To assess responses to voided urine, clitoral gland extract, or a combination of these, 50 μL test stimulus or control water was streaked onto a 55-mm glass microfiber filter stuck onto a clean petri dish lid with double-sided adhesive and the same procedure followed. Individual subject rats were introduced into the center of the arena, midway between the two stimulus tunnels for a 10-min trial, video recorded remotely using a Genie Closed-Circuit Television (CCTV) digital video recorder in a neighboring laboratory with no experimenter present in the test room. After each trial, arenas, tunnels, and mesh caps were cleaned thoroughly with Teepol and 70% ethanol.

Naturally deposited scent stimuli were gained by placing individual females into a clean arena (60 × 70 cm) containing several upturned petri dishes and left for 20 min. Petri dishes marked with visible scents were used in tests within 2 h of scent deposition. Once a test was completed, scent marks were recovered from the stimulus dish by swabbing with 50 μL PBS on a cotton bud, which was then centrifuged at 7,000*g* for 2 min to collect elution. Scent marks were then confirmed by SDS-PAGE.

Subjects and scent donors were all from our outbred hybrid rat colony. A total of 82 captive-bred adult females and 67 captive-bred adult males aged between 2 and 13 mo were used in these tests. A small number of subjects were used in more than one test but always with different unfamiliar test stimuli and with a minimum of 2 wk between successive tests [female subjects: 4/82 (4.9%) used twice; male subjects: 15/67 (22.4%) used twice]. Subjects were always unfamiliar with scent donors. Video transcription was carried out blind to the relative position of the two stimuli during each trial, which were marked as left and right. Time spent sniffing and gnawing at each of the mesh caps covering the stimulus tubes was recorded. Results from the behavioral tests are included in Dataset S4.

### Statistical Analysis of Behavioral Assays.

Analyses were carried out using R version 4.2.0 (78) with the lme4 (79) and emmeans (80) packages for mixed-effects models and IBM SPSS version 27 (IBM Ltd.) for repeated-measures ANOVAs. Binomial generalized linear mixed-effects models assessed the fixed effects of subject sexual receptivity (proestrus/estrus or metestrus/diestrus), trial type (urine stimulus or control), and donor sex and strain (same or different) on the likelihood that female rats deposited scent marks, with subject identity included as a random factor. After first examining whether females responded differently to male vs. female urine stimuli (fixed effects: subject receptivity, trial type, and donor sex; Table 1a), we ran separate models to examine responses to male and female stimuli separately, including the influence of donor strain (fixed effects: subject receptivity, trial type, and donor strain; Table 1b and c). The significance of each fixed factor and interactions between factors was compared by ANOVA to the same model with the specific factor or interaction dropped using a likelihood ratio test.

To examine attraction to female scent marks, a repeated-measures ANOVA compared the duration of investigation of the female scent stimulus vs. control as a within-trial factor (sniffing through or gnawing the covering mesh), with subject sex, donor sexual receptivity (proestrus/estrus or metestrus/diestrus), and contact with the stimulus (contact or airborne) as between-trial factors (Table 2c). As males and females responded differently according to donor sexual receptivity and contact, we ran separate models to assess the effects of donor receptivity and contact on attraction to female scent for each sex separately (Table 2a and b). Similar analyses examined male and female attraction to urinary components, clitoral secretion components, or an equal blend of these (Table 3). The distribution of residuals from each model was checked for approximation to normality by plotting and a Shapiro–Wilks test. To meet assumptions of normality, data were log transformed for models of female response and models comparing female and male responses.

### Identification of Proteins in Denaturing Polyacrylamide Gel Electrophoresis (SDS-PAGE) Bands.

Peptide map fingerprint (PMF) was performed as previously described (30). Protein maps were generated using PeptideMapper (81).

### Global Proteomic Analysis of Biological Samples.

Scent marks, urine, or clitoral gland extracts were diluted in 25 mM ammonium bicarbonate according to protein complexity and concentration. Protein concentration was measured using the Bradford method (82). Proteins in the diluted samples were then digested as described in ref. 30. Peptides were resolved by liquid chromatography [UltiMate 3000 LC nano system (Thermo Scientific)] coupled to a QExactive quadrupole orbitrap instrument (Thermo Scientific) or QExactive HF quadrupole orbitrap instrument (Thermo Scientific). Protein digests were loaded on a trap column (Acclaim PepMap 100 C18 precolumn, 300-µm id, 5-mm-long, 5-µm particles, 100 Å, Thermo Scientific) at a flow rate of 4 µL/min in 2% (v/v) acetonitrile (ACN) /0.1% (v/v) Trifluoroacetic acid (TFA). After 7 min, peptides were separated through an analytical column (Easy-spray PepMap RSLC C18 column, 75-µm id, 50-cm-long, 2-µm particles, 100 Å, Thermo Scientific) at 300 nL/min over a linear gradient from 3 to 40% (v/v) ACN in 0.1% v/v formic acid. The instruments were operated in data-dependent acquisition mode. Full scan MS spectra (m/z 300 to 2,000) were acquired at 70,000 resolution (Automatic Gain Control (AGC) set to 1e^6^ ions with a maximum fill time of 250 ms), and the 10 most intense multiply charged ions (z ≥ 2) were sequentially isolated and fragmented by high-energy collisional dissociation at 30% standardized collision energy. Fragments ions were detected at 35,000 resolution (AGC set to 1e^5^ ions with a maximum fill time of 100 ms). Dynamic exclusion was set as 20 s.

Proteome Discoverer version 1.4 (Thermo Scientific) was used to generate peak lists from raw data using default parameters. Mascot version 2.8 was used as a search engine to identify peptides and proteins. For identifications, we search our data against a database containing all entries for *R. norvegicus* in UniProt (www.uniprot.org, downloaded on 02.06.2021; 29,934 entries). Trypsin was selected as the specific enzyme allowing one missed cleavage. Carbamidomethylation of cysteine residues was set as fixed modification and methionine oxidation as variable modification, allowing a mass tolerance of 10 ppm for precursors and 0.01 Da for fragment ions. To accept an identification, we set FDR<1% at the peptide level and required at least two unique peptides identified with a MASCOT score higher than 20. Label-free quantification was performed using Progenesis QI (Waters). Since protein concentration and sample complexity differed greatly among samples, we set “normalization off” in Progenesis QI and calculated the proportional abundance of proteins. For the Mascot search, only the top five spectra were exported. Evidence for MUP truncation was derived using PEAKS v8 (Bioinformatics solutions Inc.). Proteomics data were visualized using R (v.4.2.0) (78). The mass spectrometry proteomics data have been deposited to the ProteomeXchange Consortium via the PRoteomics IDEntification (PRIDE) (83) partner repository with the dataset identifier PXD039325 and 10.6019/PXD039325.

### ESI-MS of Intact Proteins.

Samples were diluted in 0.1% (v/v) formic acid and centrifuged at 13,000*g* for 10 min. All analyses were performed on a Synapt G2 mass spectrometer (Waters Corporation), fitted with an Atmospheric Pressure Ionization (API) source. Samples were desalted and concentrated on a C4 reverse phase trap (Thermo Scientific), and protein was eluted at a flow rate of 10 μL/min using three repeated 0 to 100% acetonitrile gradients. Data were collected between 800 and 3,500 Th (m/z), processed, and transformed to a neutral average mass using MaxEnt 1 (maximum entropy software, Waters Corporation). The instrument was calibrated with 250 fmol of myoglobin (16.7 kDa) from equine heart (Sigma-Aldrich).

## Supplementary Material

Appendix 01 (PDF)

Dataset S01 (XLSX)

Dataset S02 (XLSX)

Dataset S03 (XLSX)

Dataset S04 (XLSX)

## Data Availability

Proteomics data have been deposited in ProteomExchange (PXD039325) (84).
