# Supplementary material for: Unraveling female communication through scent marks in the Norway rat
Source: Proc Natl Acad Sci U S A. 2023 Jun 12;120(25):e2300794120. doi: 10.1073/pnas.2300794120 (PMC10288631; doi:10.1073/pnas.2300794120)
Supplement: Supplementary file 1 — Appendix 01 (PDF) [file pnas.2300794120.sapp.pdf]

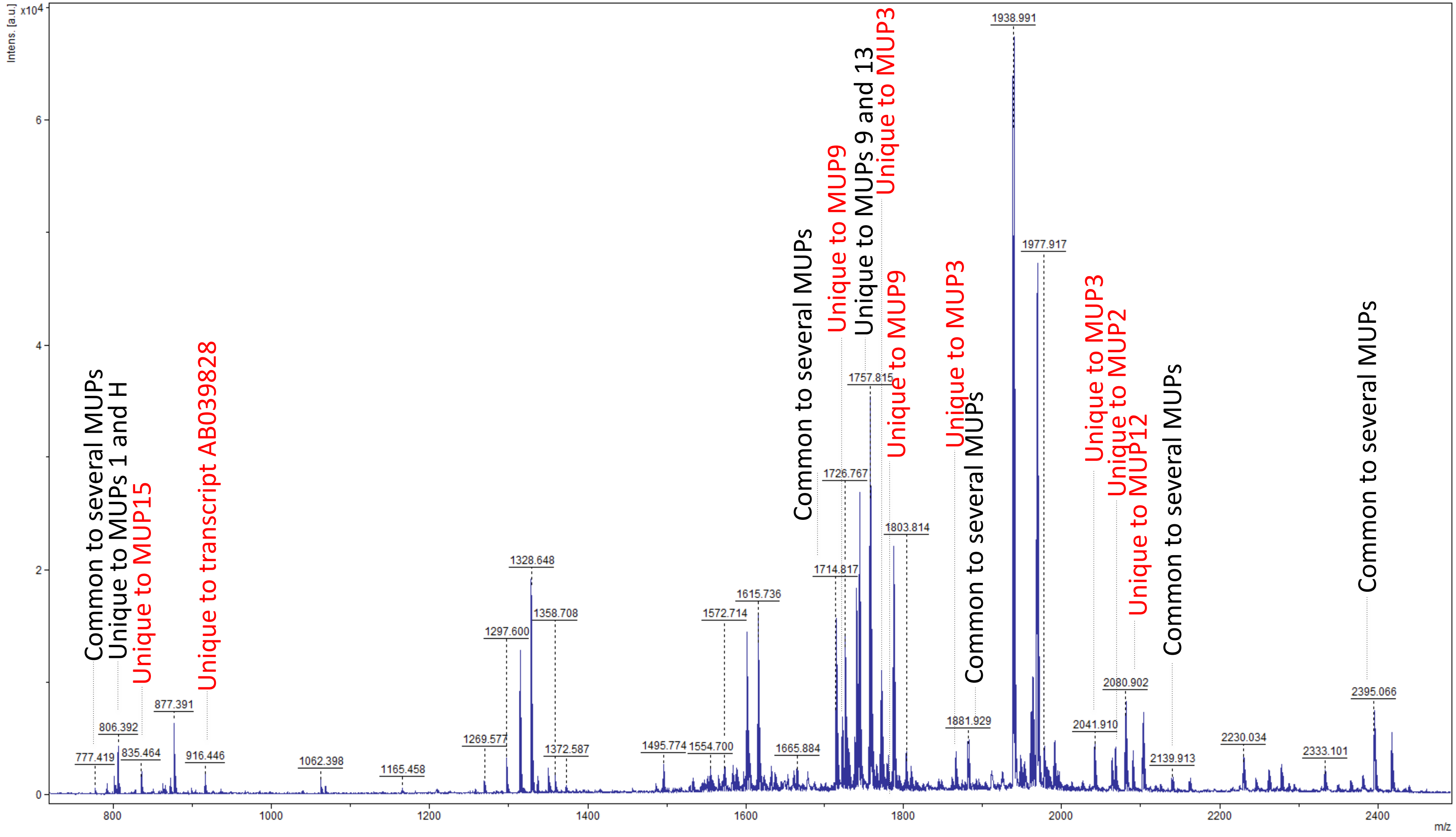

Supplementary Figure 1: Peptide mass fingerprinting of the 17kDa band in female rat scent marks. The band was excised from an SDS-PAGE gel and digested with endopeptidase Lys. Peptides of the same mass as known MUP LysC fragments are labelled.

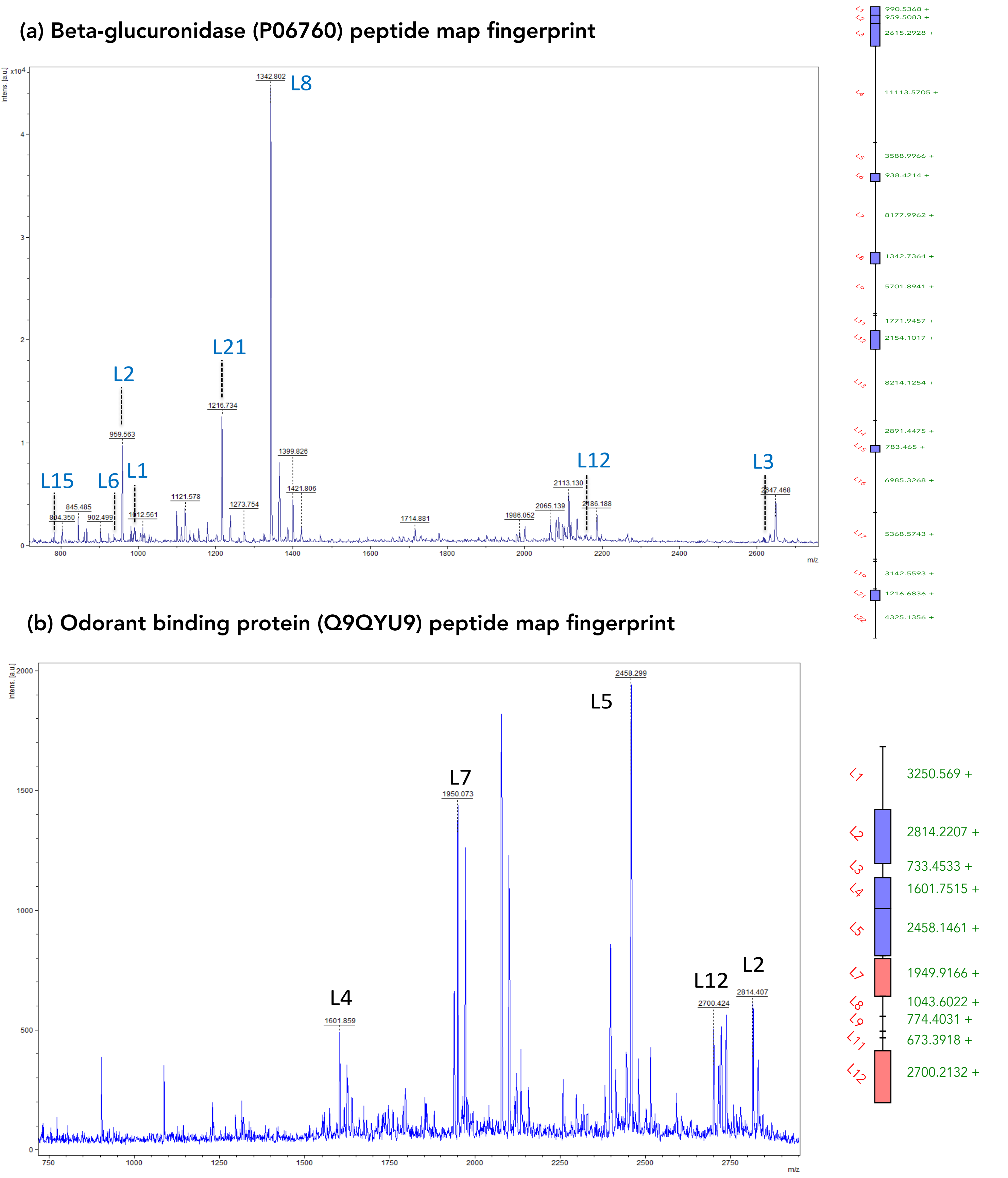

Supplementary Figure 2: Peptide mass fingerprinting of the 80 kDa and 20 kDa bands in female rat scent marks. The bands (80 kDa (panel a) and 20 kDa (panel b)) were excised from a SDS-PAGE gel and protein digested with endopeptidase Lys C. The reconciliation of mass peaks with the protein sequences was used to create peptide maps (right had side of the spectrum). PMF determined that the 80 kDa band corresponded to beta-glucuronidase. There are two different sequences in UniProt for rat b-glucuronidase (UniProt KB P06760 and F1LQQ8) of the same length but with three sequence conflicts at positions 14, 21 and 487. It was not possible to discriminate between these two variants because two of these positions are located within the signal peptide (positions 14 and 21) and thus absent from the mature secreted form of the protein, while position 487 lies in very large peptides for either trypsin or LysC digestion (peptide T43 predicted mass 5773 Da; peptide L16 predicted mass 6927 Da), which were not visible in these analyses. PMF determined that the 20 kDa band corresponded to odorant binding protein Q9QYU9. Red boxes represent unique peptides for this protein, blue boxed represent peptides shared with the odorant binding protein 1f (P08937).

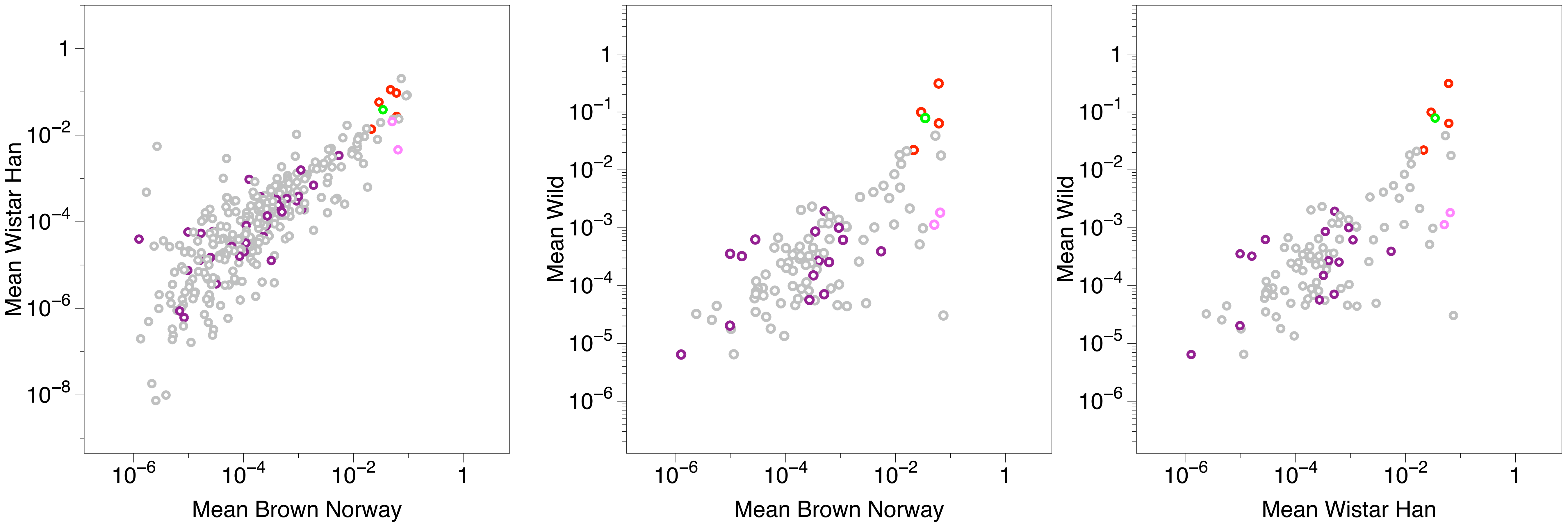

Supplementary Figure 3: Protein abundance correlations between different strains used and wild female individuals. Female rat scent marks were recovered by swabbing from Brown Norway (BN, n=7), Wistar Han (WH, n=9) and wild rats (Wild, n=8) and analysed by label-free quantitative proteomics. For proteins common to each source, the label free abundances were plotted to reveal an overall quantitatively similar profile in scent marks from each source. Red: rat MUP isoforms; green: β-glucuronidase; purple: proteolysis related proteins; pink: rat serum albumin.

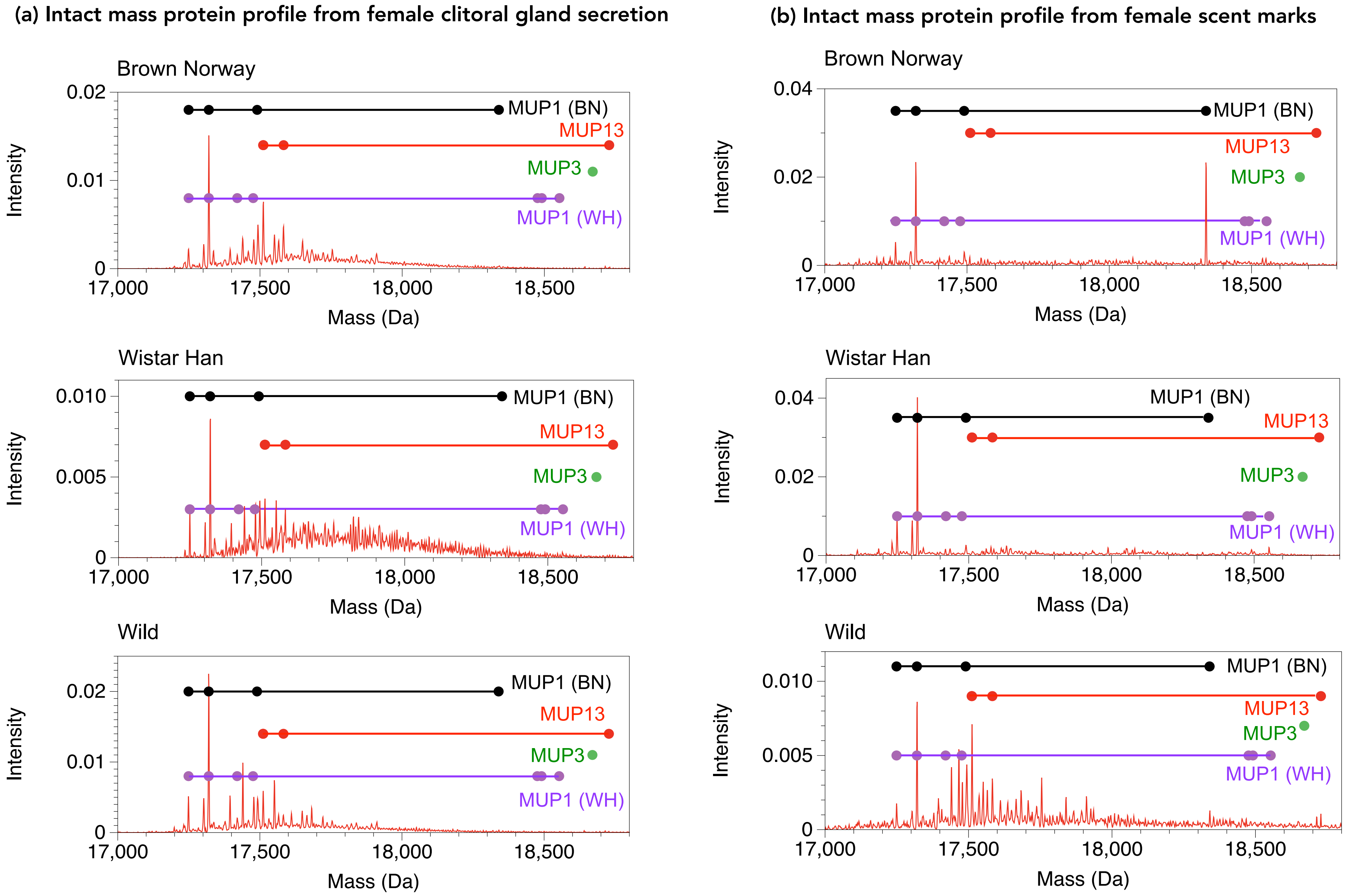

Supplementary Figure 4: Evidence of proteolytic trimming in clitoral gland extracts and scent marks. Electrospray ionisation mass spectrometry of the clitoral gland secretion reveals multiple masses, many of which can be reconciled by N and C terminal trimming of MUPs that are present in this secretion, highlighted by sequences of dots on a line representing one MUP (a). A similar pattern of trimming is evidence in scent marks (b).

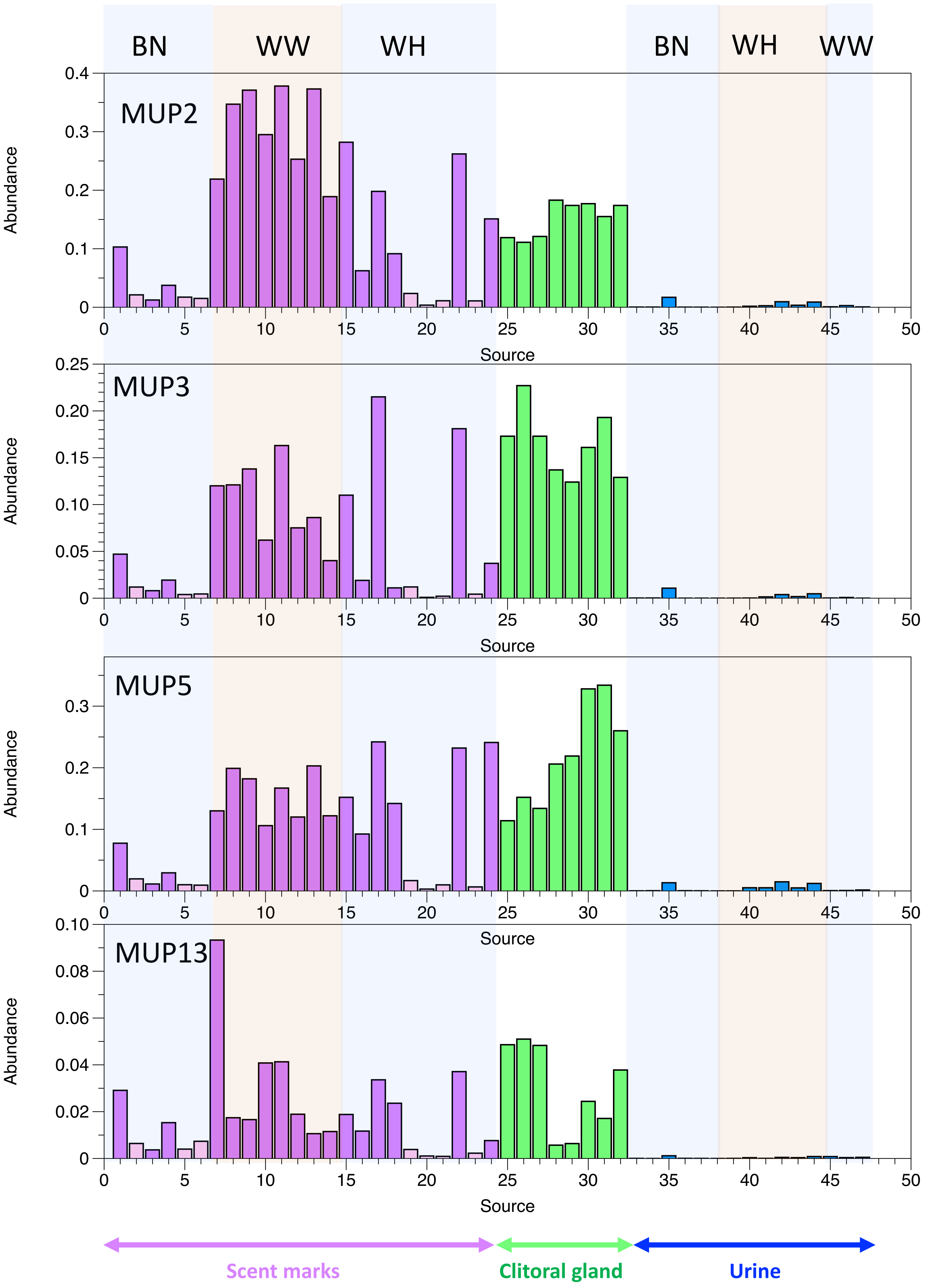

BN
WW
WH
BN
WH
WW
MUP2
MUP3
MUP5
MUP13
Abundance
Source
Scent marks
Clitoral gland
Urine

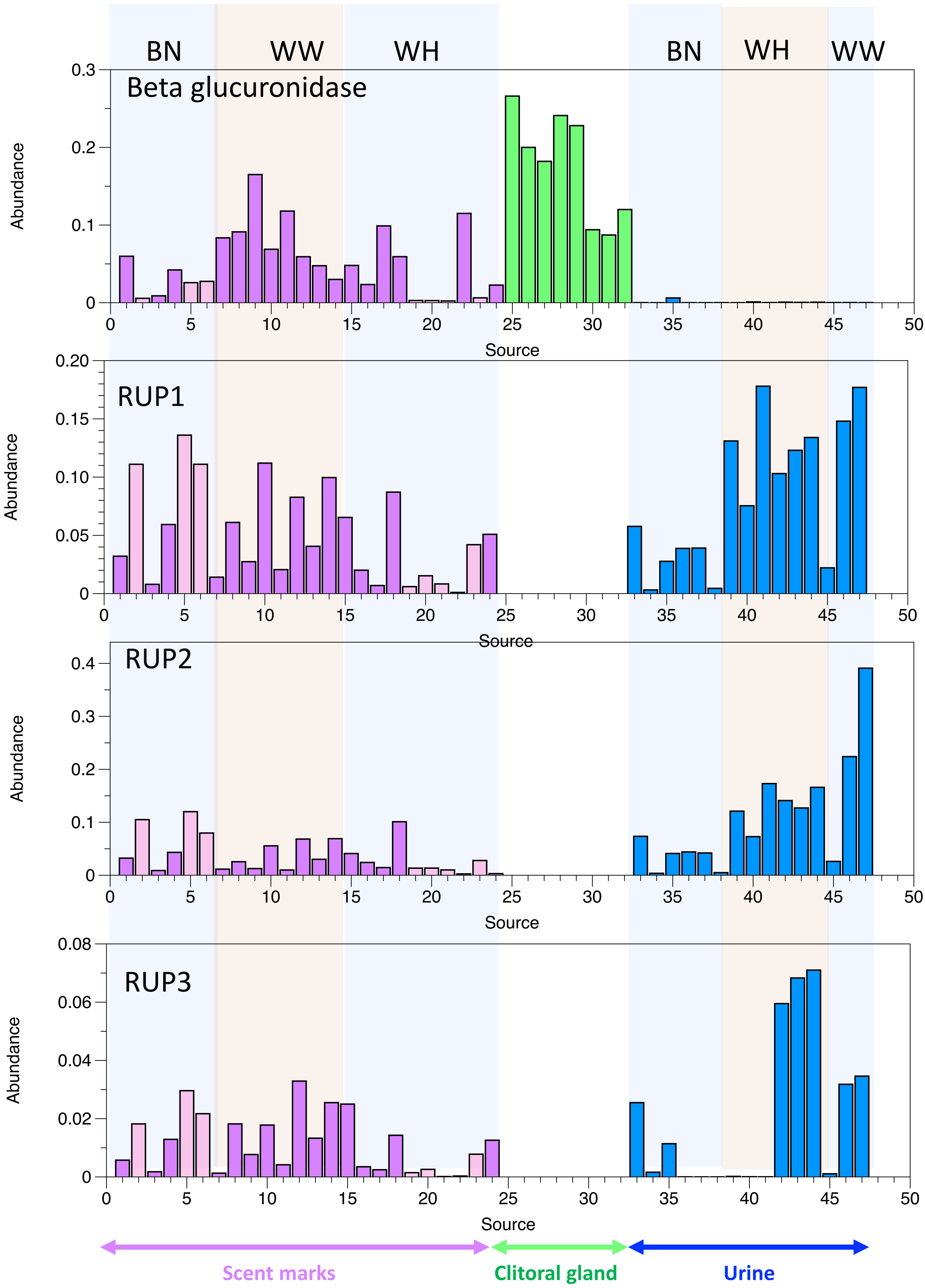

BN
WW
WH
BN
WH
WW
Beta glucuronidase
RUP1
RUP2
RUP3
Abundance
Source
0.3
0.2
0.1
0
0.20
0.15
0.10
0.05
0.4
0.08
0.06
0.04
0.02
0
5
10
15
20
25
30
35
40
45
50
Scent marks
Clitoral gland
Urine

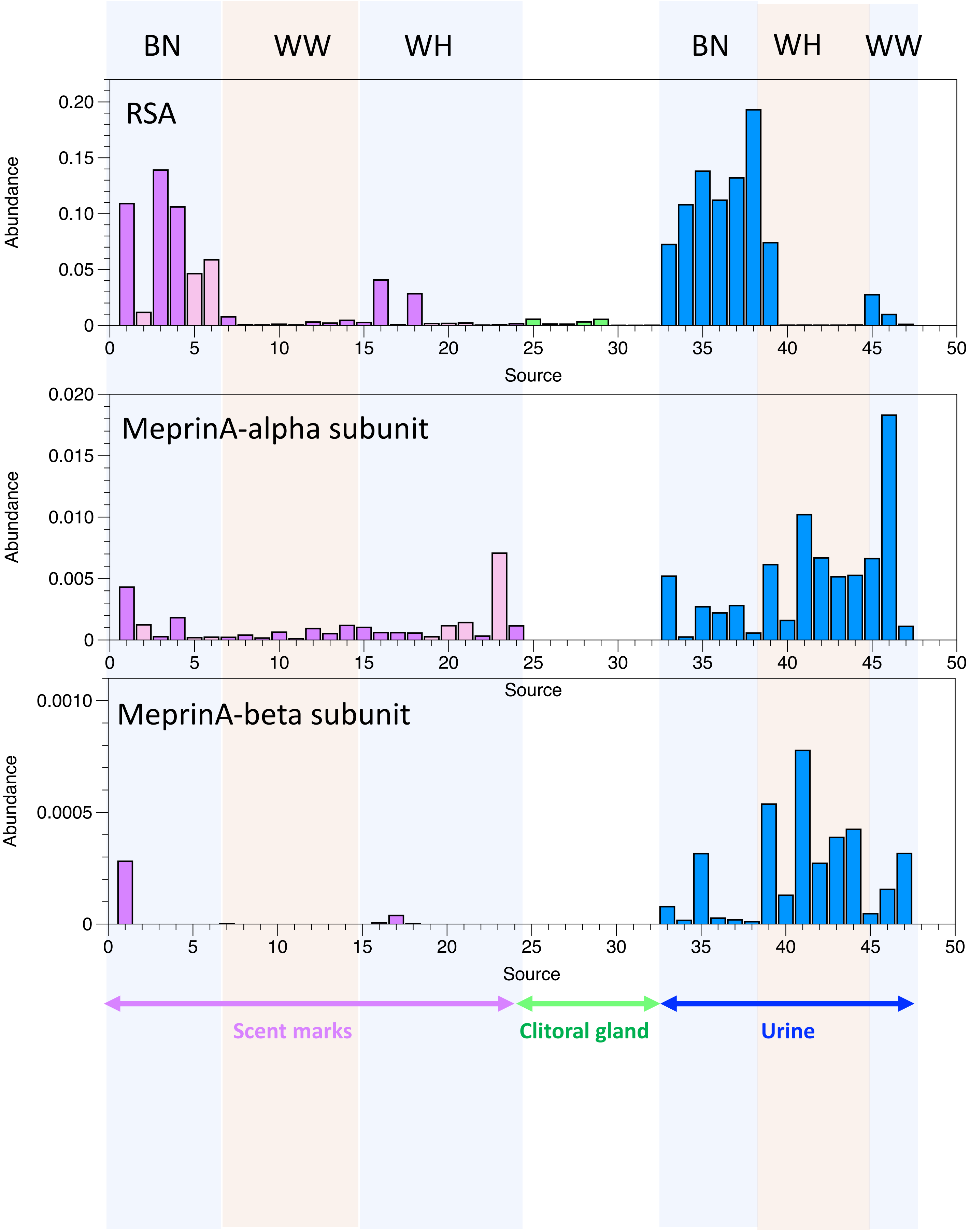
BN
WW
WH
BN
WH
WW
RSA
Abundance
Source
MeprinA-alpha subunit
Abundance
Source
MeprinA-beta subunit
Abundance
Source
Scent marks
Clitoral gland
Urine

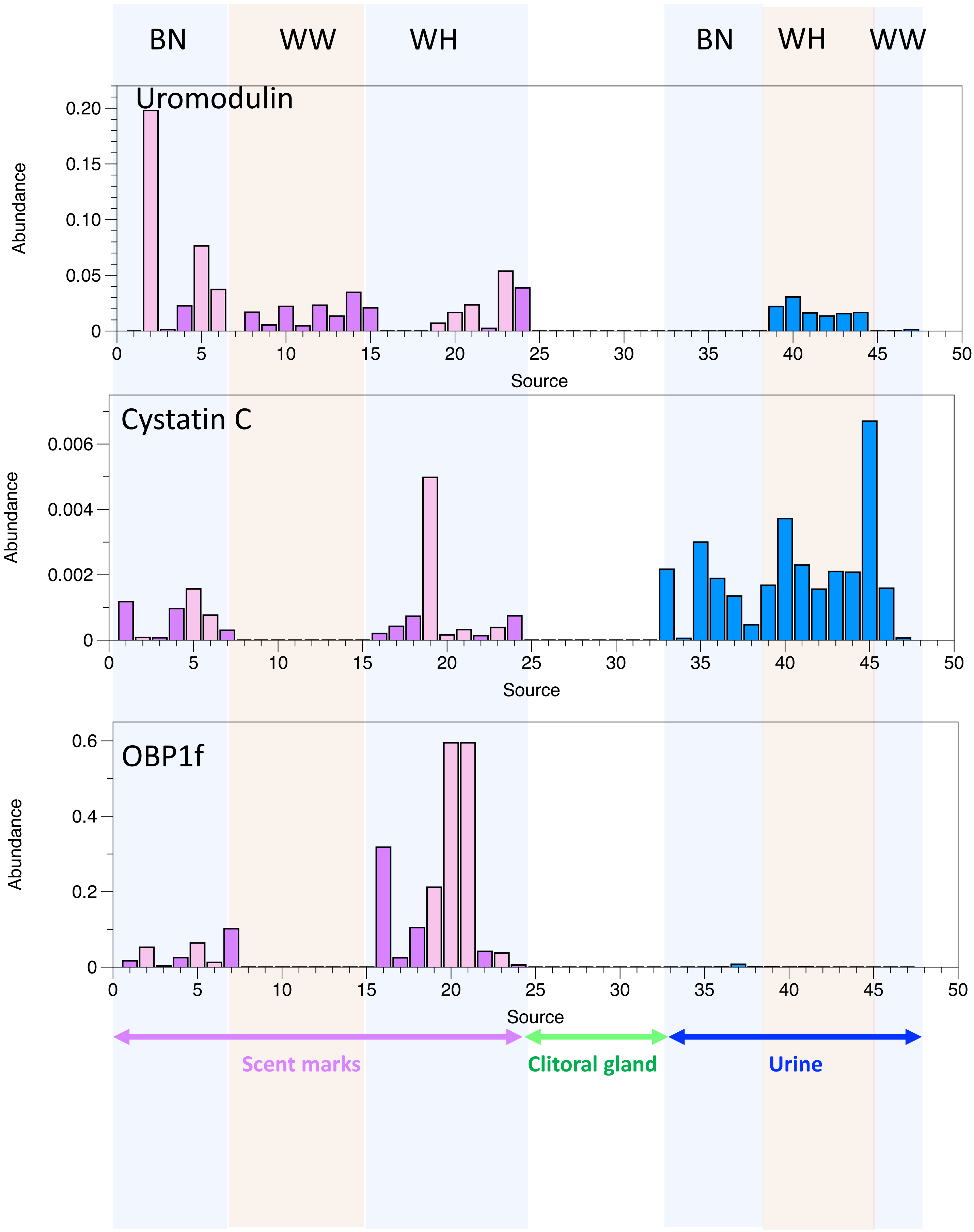
BN
WW
WH
BN
WH
WW
Uromodulin
Abundance
Source
Cystatin C
Abundance
Source
OBP1f
Abundance
Source
Scent marks
Clitoral gland
Urine

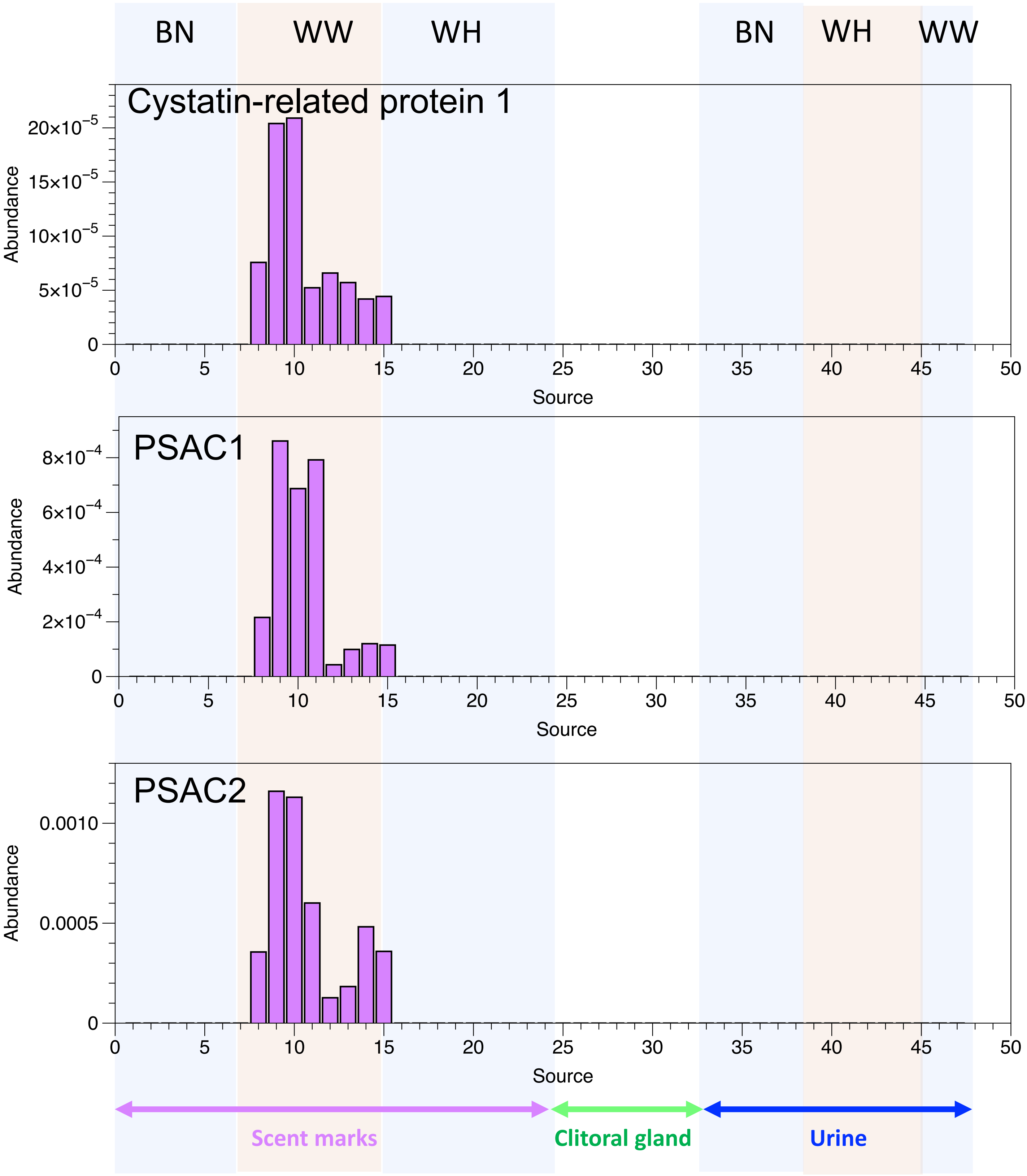

Supplementary Figure 5: Specific protein profiles in scents and scent components. The relative abundances of a series of proteins were measured by label free proteomics across a series of scent marks (pink/purple), clitoral gland secretions (green) and urine (blue) collected from Wistar Han, Brown Norway and wild rats. Scent marks were further flagged as having a predominant 17kDa 'mup-like' band on SDS page (purple) or not (pink). MUPs and beta glucuronidase are consistent with clitoral gland contribution to scents. Rat serum albumin, Rat Urinary Proteins (RUPs), uromodulin and cystatin C and meprin are all representative of urinary proteins and contribution. PSAC1, PSAC2 and cystatin related protein 1 likely reflect Skene gland contribution.

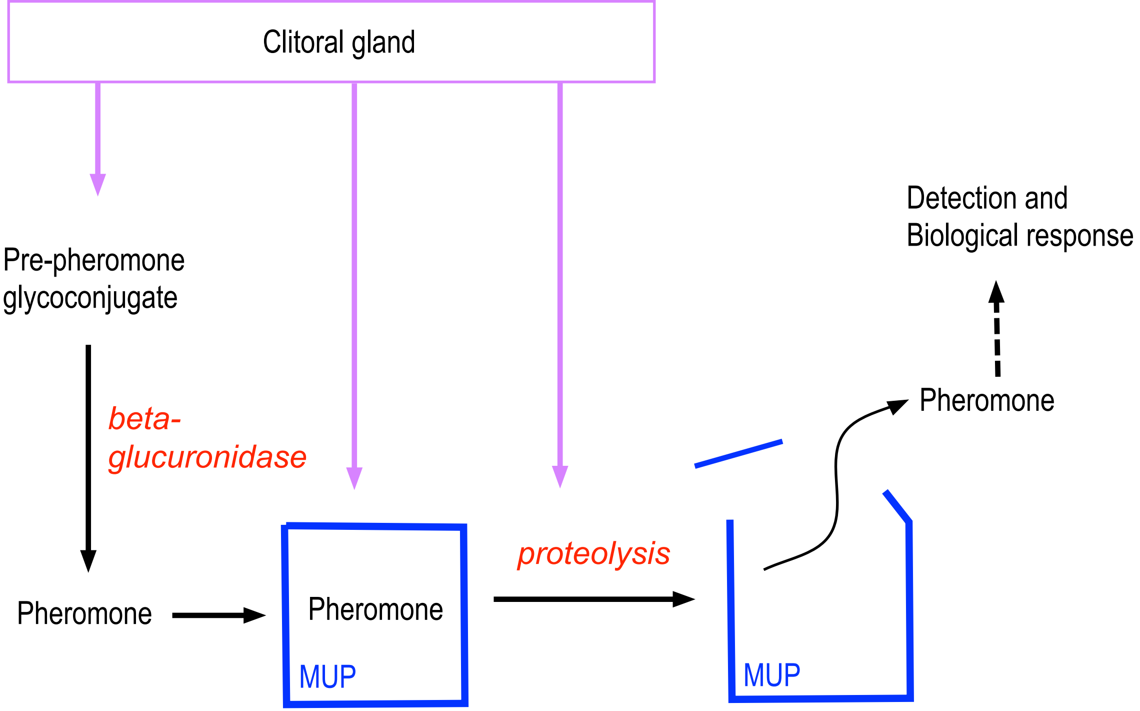

Supplementary Figure 6. A working hypothesis for elaboration of scent mark complexity in the female rat.

Legends for supplementary datasets

Dataset S1: Compiled results related to LC-MSMS identification after in-gel digestion from SDS-PAGE of the 17 kDa bands. Digests of these bands using trypsin to produce shorter peptides were analysed by LC-MS/MS. Peak lists were searched against a in house database containing all MUP mature sequences currently predicted in the rat.

Dataset S2: Compiled results related to LC-MSMS identification in female scent samples. A global proteome analysis was performed on scent deposit samples. A total of 469 proteins were identified at a false discovery rate of 1% and requiring at least two unique peptides identified per protein.

Dataset S3: Compiled results related to LC-MSMS identification in a comparative proteomic survey in female scent samples, urine, and clitoral secretions. A total of 930 proteins were identified at 1% false discovery rate, with a minimum of two unique peptides for identification, with a total of 506 protein groups in clitoral gland secretion, 461 protein groups in urine and 469 protein groups in scent mark samples.

Dataset S4: Results from the behavioural tests.
